# Roles of the MO25 protein Pmo25 in contractile-ring stability and localization of the NDR kinase Sid2 during cytokinesis

**DOI:** 10.1101/2025.05.13.653815

**Published:** 2025-05-14

**Authors:** Yanfang Ye, Sha Zhang, Aysha H. Osmani, Jack R. Gregory, Evelyn Goodyear, Jian-Qiu Wu

**Affiliations:** 1 Department of Molecular Genetics, The Ohio State University, Columbus, OH 43210, USA; 2 College of Agriculture, Fujian Agriculture and Forestry University, Fuzhou, 350002, China; 3 Graduate Program of Molecular, Cellular, and Developmental Biology, The Ohio State University, Columbus, OH 43210, USA; 4 Lead Contact

**Keywords:** Cdc4, contractile ring, cytokinesis, glucanase Eng1, MO25, Munc13/UNC-13, Sid2, septum, SIN pathway, Ync13

## Abstract

Mouse protein-25 (MO25) proteins are crucial in development and morphogenesis from plants to humans. Fission yeast MO25 protein Pmo25 is essential for cell polarity and division. However, how Pmo25 regulates cytokinesis remains largely unknown. We identified and confirmed that Pmo25 binds to the Munc13/UNC-1 3 family protein Ync13 through yeast two-hybrid screen, co-immunoprecipitation, and in-vitro binding assays. In *pmo25* mutants, contractile-ring stability and septation are defective during cytokinesis; the rings slide on the plasma membrane and some disintegrate before or during constriction. Moreover, Pmo25 interacts directly with the myosin-II light chain Cdc4, which is essential for the contractile-ring assembly and functions. Furthermore, division-site recruitment of the NDR kinase Sid2 in the Septation Initiation Network and the glucanase Eng1 for daughter-cell separation are compromised in *pmo25* mutants, consistent with the cytokinesis defects. Our findings reveal a novel mechanism on how Pmo25 regulates cytokinesis and suggest that conserved MO25 proteins can link various cellular processes.

## INTRODUCTION

Cell division and cell morphogenesis are fundamental determinants for cell proliferation, differentiation, and development. The cell cycle transitions must be regulated coordinately to ensure that these processes proceed properly. This raises the question of how cells maintain the dynamic transition of interconnected signaling networks and signal fidelity. The rod-shaped fission yeast cells grow from cell ends and divide by medial fission. The mechanisms and signaling cascades that control the growth and division are highly conserved in various aspects from yeasts to higher eukaryotes ^1-7^. Thus, fission yeast is an ideal genetically tractable model system to elucidate these conserved mechanisms and signaling pathways.

Cytokinesis is the last step of the cell-division cycle, which partitions cytoplasm and organelles from a mother cell to two daughter cells. In most eukaryotic cells from fungi to mammalian cells, cytokinesis occurs with several crucial steps: cleavage site selection; actomyosin contractile ring assembly, maturation, constriction; plasma membrane deposition and expansion at the division plane; and extracellular matrix formation or remodeling ^8-12^. Cytokinesis also needs the cooperation of signaling pathways and membrane trafficking, including exocytosis and endocytosis, to succeed ^13-18^. The Septation Initiation Network (SIN) mediates growth at the division site and cytokinesis in the fission yeast *Schizosaccharomyces pombe*. The SIN pathway resembles the Mitotic Exit Network in budding yeast and the mammalian Hippo pathways ^3,5,19-21^. It is essential for contractile-ring assembly and maintenance and also for cell growth at the division site to form the septum and new cell ends ^22-24^. The signaling of the SIN pathway is controlled by the GTPase Spg1, which in turn activates three downstream kinases: Cdc7, Sid1, and Sid2 (an NDR kinase). Except for Sid2, most SIN components are situated at the spindle pole body (SPB) ^25,26^, which is functionally equivalent to centrosomes. Upon activation, the Sid2-Mob1 complex translocates from the SPBs to the medial division site where it facilitates contractile ring assembly and constriction as well as septum formation ^27^. Failure of SIN signaling causes cytokinesis defects; however, cells continue the growth and nuclear cycle, resulting in elongated and multinucleated cells before cell lysis. It has been reported that the SIN inhibits the Morphogenesis Orb6 Network (MOR) signaling pathway in mitosis by interfering with Nak1-mediated activation of the most downstream MOR component, Orb6, which is the other NDR kinase in fission yeast ^1,28,29^. MOR signaling regulates actin assembly and is essential for cell polarity and morphogenesis ^30-33^. In addition, Cdc7 and Sid1 kinases also control the MOR components, the localization of Pmo25 (MO25 protein) at the SPBs, and Nak1-Orb6’s kinase activities during interphase ^33,34^.

MO25 was originally identified as a gene expressed at the early cleavage stage of mouse embryogenesis ^35^. MO25 is a scaffold protein that forms a heterotrimeric complex with the pseudokinase STE20-related adaptor (STRAD) and the tumor suppressor liver kinase 1 (LKB1). The LKB1-STRAD-MO25 complex stabilizes a closed conformation of STRAD and triggers LKB1 nucleocytoplasmic shuttling and activation ^36-39^. LKB1 modulates several cellular processes, such as cell growth and polarity, through downstream AMP-activated protein kinase (AMPK) and AMPK-related kinases (ARKs) ^40,41^. Besides the role in the LKB1-STRAD-MO25 complex, MO25 is a key regulator of the SPAK/OSR1 and MST3/MST4/YSK1 protein kinases as well, which regulate ion homeostasis/blood pressure and development/morphogenesis, respectively ^42^.

MO25 proteins are highly conserved in all eukaryotes from plants to humans ^43-46^. In budding yeast, the MO25-like protein Hym1 functions in the Regulation of Ace2 and Morphogenesis (RAM) network, which is equivalent to the MOR in *S. pombe* and is essential for cell polarity, morphogenesis, and daughter-cell separation ^47,48^. It has been proposed that RAM signaling regulates both localization and activation of transcription factor Ace2 through Cbk1 (Orb6 orthologue) phosphorylation ^49,50^. Hym1 allosterically activates the Hippo-like kinase, Kic1 (Nak1 orthologue), which in turn phosphorylates Cbk1 ^4^. In addition, Hym1 is involved in the G1 to S phase transition ^51^. In *Neurospora crassa*, HYM1/MO25 controls the cell morphogenesis NDR kinase module as well as MAP kinase signaling during intercellular communication ^51^. Similarly, Pmo25, a component of the MOR network in fission yeast, forms a complex with Nak1 and is required for Nak1 localization and kinase activity, and both Pmo25 and Nak1 are essential for Orb6 activation ^33,34,52^. Pmo25 functions as an upstream regulator of the MOR network and mediates signaling connection between the SIN and MOR ^33,34,52^. Pmo25 has also been reported to interact with GCK kinase Ppk11 ^53^. *pmo25* mutants show defects in cell morphogenesis and separation ^33,34,52^. However, unlike its roles in cell morphogenesis, the molecular mechanism on how Pmo25 regulates cell separation during cytokinesis remains unknown.

Ync13 is a member of the Munc13/UNC-13 protein family, which is conserved in plants, fungi, and humans ^54-58^. It is essential for cytokinesis in fission yeast grown in rich medium ^58^. UNC-13/Munc13 proteins have conserved functions in vesicle priming and tethering before vesicle fusion with the plasma membrane during exocytosis ^54-57,59-65^. Ync13 localizes to cell tips during interphase and is recruited to the division site during early anaphase and stays there until cell separation ^58^. Ync13 modulates the recruitment, maintenance, and spatial distribution of cell wall enzymes including the glucan synthases Bgs1, Bgs4, Ags1, and the glucanase Eng1 during cytokinesis ^58,66-77^. Although there are no obvious defects in early cytokinesis, *ync13* mutants are defective in septum formation, which results in cell lysis during cell separation ^58^. However, the functions of Ync13 in cytokinesis remain poorly understood.

In this study, we identify Pmo25 as a binding partner of Ync13 in a yeast two hybrid screen and provide evidence that Pmo25 regulates Ync13 and Eng1 recruitment to the division site. Our data further suggest that Pmo25 is not only required for cell separation in late cytokinesis but also for contractile ring stability. Interestingly, Pmo25 interacts with the myosin-II essential light chain Cdc4 and the SIN kinase Sid2, and Pmo25 is important for Sid2 localization to the division site. Collectively, our data suggest that Pmo25 serves as a master scaffold that links several signaling pathways to coordinate cellular processes and inter-signaling transduction.

## RESULTS

### Pmo25 is a binding partner of Ync13

**Figure 1.**
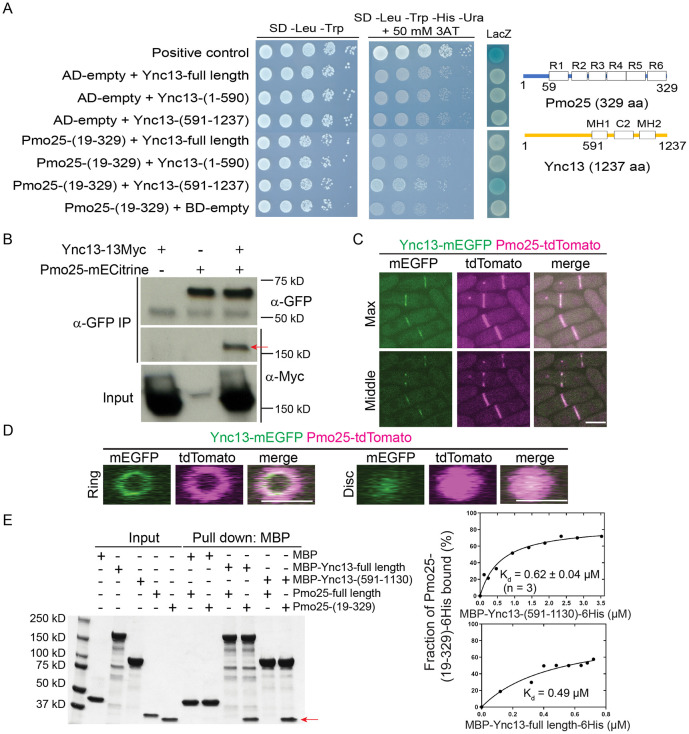
The Munc13/UNC-13 protein Ync13 interacts with the MO25 protein Pmo25. (A) Interaction between Ync13 and Pmo25 in yeast two hybrid assays. Left, different combinations of AD-Pmo25 and BD-Ync13 fragments were transformed into *S. cerevisiae* MaV203 cells. Interaction was detected by 10x serial dilution on quadruple dropout medium with 3-aminotriazole (3AT) and LacZ coloration. Right, Domain structures of Pmo25 and Ync13 ^33,45,58,128^. Pmo25 has six armadillo-like helical repeats (R1-R6). Ync13 has C2 domain and two Munc13 homology domains (MH). Numbers indicate the aa. (B) Co-IP of Ync13 and Pmo25. Cell extracts were immunoprecipitated with anti-GFP antibody. Precipitated and input samples were then detected using monoclonal antibodies against Myc and/or GFP. (C and D) Colocalization of Pmo25 and Ync13 in max projection and middle focal plane (C) and 3D projection (D). Cells (JW7970) expressing Pmo25-tdTomato and Ync13-mEGFP were grown at 25°C for ~36 h before imaging. Maximal intensity projection of 16 slices with 0.4 μm spacing (C) and 3D projections (D) are shown. Bars, 5 μm. (E) In vitro binding assay of Ync13 and Pmo25. Ync13 full length (1237 aa), Ync13 C terminal 591-1130 aa, Pmo25 full length (329 aa), and Pmo25 C terminal 19-329 aa were purified from *E. coli*. Binding assay was performed with MBP pull down. Left, protein samples analyzed by SDS-PAGE and stained with Coomassie Blue, the arrow marks the Pmo25-(19-329) band. Right, the curve fits and *K_d_* values for Pmo25-(19-329) with Ync13-(591-1130) and Ync13-full length, respectively.

The Munc13/UNC-13 protein Ync13 in the fission yeast *S. pombe* plays a critical role in the cell-wall integrity during cytokinesis, but its binding partners were unknown ^58^. To identify Ync13’s binding partners, we carried out yeast-two-hybrid screens. As full length Ync13 showed high levels of auto-activation in the *HIS3* and *URA3* reporter assays, Ync13 NH_2_- (1-590 aa) and COOH-terminus (591-1237 aa) were used as baits. A (19-329)-aa fragment of Pmo25 (SPAC1834.06c, full length: 329 aa) was found to exhibit positive interaction with Ync13 COOH-terminus (591-1237 aa), but not with its NH2-terminus (1-590 aa) (Figure 1A).

To confirm the Pmo25-Ync13 interaction in *S. pombe* cells, we tagged Pmo25 and Ync13 at their COOH-terminus with mECitrine and 13Myc, respectively. After immunoprecipitating Pmo25 with anti-GFP antibody, Ync13-13Myc was pulled down from the cells co-expressing Pmo25-mECitrine and Ync13-13Myc but not in the single tagged cells, indicating that Pmo25 and Ync13 interact in fission yeast cells (Figure 1B). In addition, Ync13-mEGFP and Pmo25-tdTomato colocalized at the division site, though Ync13 was more concentrated at the leading edge of the cleavage furrow (Figure 1, C and D), which was confirmed using cells expressing Ync13-mECitrine and Pmo25-mScarlet. But only Pmo25 localized to the SPBs (Figure 1C). To further confirm whether Pmo25 interacts with Ync13 directly, we purified MBP-tagged full length Ync13 and Ync13 COOH-terminus (591-1130 aa), and His-tagged Pmo25 full length and Pmo25 COOH-terminus (19-329 aa) from *E. coli*. In vitro binding assays showed that neither Ync13 full length nor Ync13 C-terminus (591-1130 aa) interacted with Pmo25 full length, but both interacted with Pmo25-(19-329 aa), with *K_d_* values of 0.49 μM and 0.62 μM respectively (Figure 1E). Together, these results indicate that Pmo25 is a direct interactor of Ync13 in fission yeast.

### Pmo25 affects Ync13 accumulation at the division site

**Figure 2.**
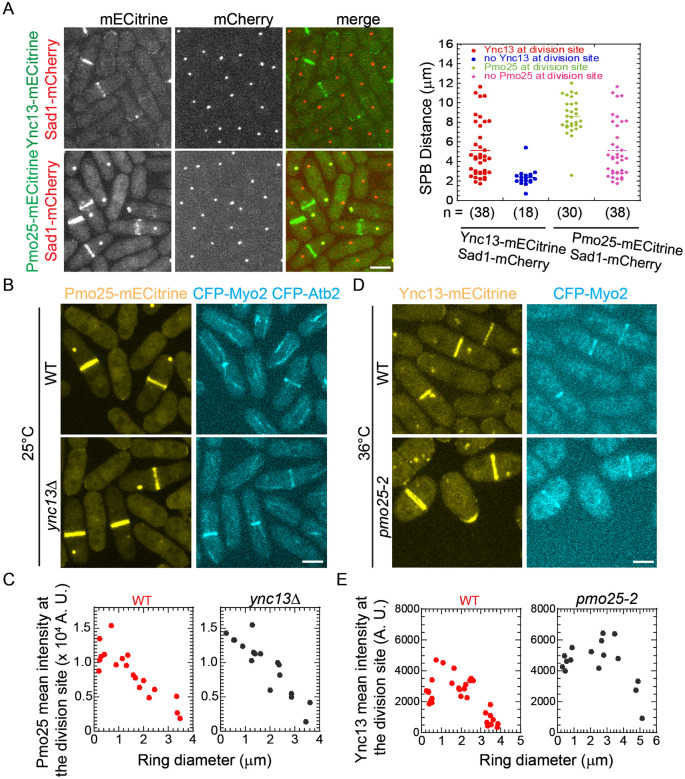
Timing of Pmo25 localization to the division site and its effect on Ync13 level during cell division. (A) Micrographs (left) and quantifications (right) of Pmo25 (JW7968) and Ync13 (JW5814) localizations in the cells with Sad1-mCherry as a cell-cycle marker. Right, the timing of Pmo25 and Ync13 appearance at the division site relative to the distance between two SPBs. (B and C) Localization and division site intensity of Pmo25 in WT and *ync13Δ* mutant. Cells of WT (JW8174) and *ync13Δ* mutant (JW8396) were grown in YE5S + 1.2 M sorbitol at 25°C for ~36 h, then washed into YE5S and grown for 3 h before imaging. The division site intensity of Pmo25 was measured in SUM projection and plotted versus Myo2 ring diameter. (D and E) Localization and division site intensity Ync13 in WT and *pmo25-2* mutant. Cells of WT (JW8135) and *pmo25-2* mutant (JW8136) were grown at 25°C for ~36 h, then shifted to 36°C for 2 h before imaging. The division site intensity of Ync13 was measured in SUM projection and plotted versus Myo2 ring diameter.

To determine the functional relationship between Pmo25 and Ync13, we tested their localization dependency. Ync13-mECitrine localized to the cell tips in interphase and the division site during mitosis and cytokinesis; and Pmo25-mECitrine localized to the SPB and the division site during mitosis and cytokinesis (Figure 2, A-D), consisted with previous reports ^33,34,58^. When the SPB marker Sad1-mCherry was used to monitor mitotic progress, >67% of cells with two SPBs showed Ync13 localization at the division site, and the mean SPB distance was ~5 μm. In contrast, <50% of cells with two SPBs showed Pmo25 localization at the division site, and the mean SPB distance was ~8 μm in cells with Pmo25 at the division site, indicating that Ync13 appears at the division site earlier than Pmo25 (Figure 2A). However, *ync13Δ* cells had no obvious defect in Pmo25 localization at the division site (Figure 2, B and C). Deletion of *ync13* leads to cell lysis in YE5S rich medium without the osmotic stabilizer sorbitol ^58^. Pmo25 localized to the SPBs and the division site at comparable levels in *ync13Δ* cells and WT cells (Figure 2, B and C). However, although Ync13 still localizes to the cell tips and the division site in *pmo25-2* cells at the restrictive temperature 36°C, its intensity increased significantly (Figure 2, D and E). Thus, Pmo25 is important for Ync13 accumulation at the division site.

### Pmo25 is important for contractile-ring formation, stability, and septation during cytokinesis

**Figure 3.**
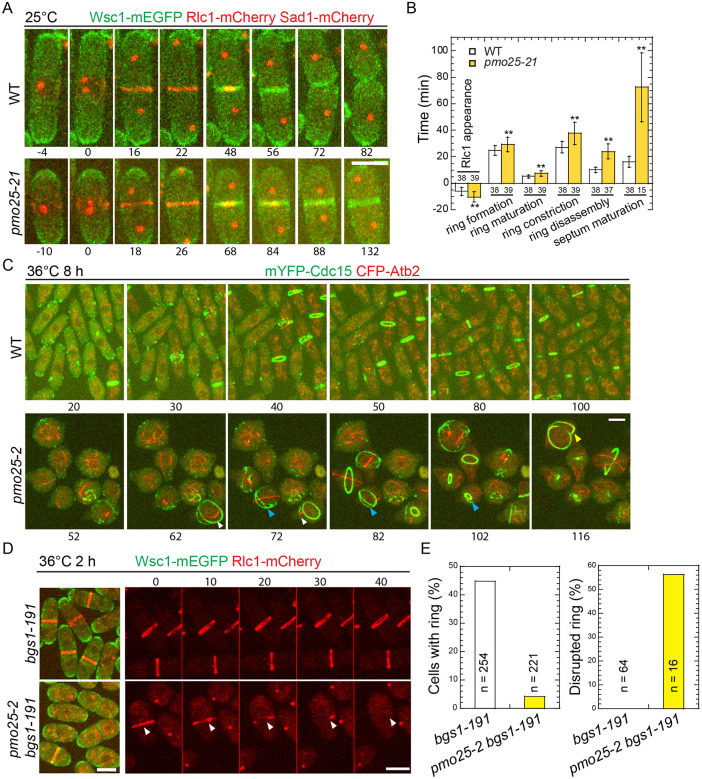
*pmo25* mutants are defective in cytokinesis. (A and B) Time course (A) and quantification (B) of the main cytokinesis events (in min) of cells expressing Wsc1-mEGFP Rlc1-mCherry Sad1-mCherry in WT (JW8614) and *pmo25-21* mutant (JW8615) at 25°C. Time 0 marks the separation of two SPBs. Numbers of cells analyzed are shown above or below the bars. **p < 0.001. (C) Time lapse (in min) of mYFP-Cdc15 CFP-Atb2 in WT (JW3186) and *pmo25-2* mutant (JW8148). Cells were grown in log phase at 25°C for ~36 h, then shifted to 36°C for 8 h before imaging at ~36°C. Times are minutes after the start point of movie. Yellow, white, and blue arrowheads indicate the same cells that formed abnormal ring during the movie. (D and E) Micrographs (D) and quantification (E) of Wsc1-mEGFP Rlc1-mCherry in *bgs1-191* (JW2766) and *pmo25-2 bgs1-191* mutant (JW8575) at 36°C. Cells were grown in log phase at 25°C for ~36 h, then shifted to 36°C for 2 h before imaging at 36°C. (D) Left, single time-point image; Right, time lapse (in min) shown Rlc1 signal only. Time 0 marks the start of the movie. Arrowheads mark a collapsed ring. (E) Left and right graphs show percentage of cells with contractile ring and ring break-down before fully constriction during the time course, respectively. Bars, 5 μm.

Despite the previous studies ^33,34,52^, the roles of Pmo25 in cytokinesis remain poorly understood. As Pmo25 is an essential gene, to further explore the functions of Pmo25 in cytokinesis, we created mutants of *pmo25* using the marker reconstitution mutagenesis method by replacement of the chromosomal *pmo25* gene with error-prone PCR products amplified using full length *pmo25*^+^ as previously described ^78-81^. *pmo25-21* showed similar polarity and septation defects as other previously characterized mutants *pmo25-2* and *pmo25-35* ^33,34^ at 36°C (Figure 3, A-C). Cells gradually lost polarity and became rounder with extended growth at 36°C (Figure 3C). Even at 25°C, *pmo25-21* cells delayed septation. We used the plasma membrane-associated serine-rich cell wall sensor Wsc1, α-tubulin Atb2, or the SPB protein Sad1, and the myosin-II regulatory light chain Rlc1 or the F-BAR protein Cdc15 to examine the plasma membrane, cell cycle stages, and contractile ring during cytokinesis, respectively (Figure 3). In WT cells, the contractile ring assembled from the precursor nodes and constricted to guide plasma membrane invagination and septum formation (Figure 3, A and B) as reported ^9,82,83^. In *pmo25-21* mutant at 25°C, Rlc1-mCherry nodes appeared at the division site ~10.5 min before SPB separation, and the compact-ring formation (from nodes appearance to condensation into a ring without lagging nodes), maturation (from a compact ring to the start of fast phase of ring constriction ^84^), constriction (from the start of fast-phase of ring constriction ^84^ to the ring constricted to a dot at cell center with highest Rlc1 pixel intensity), disassembly (from ring dot to Rlc1 disappearance from the division site), and septum maturation (from Rlc1 disappearance to the start of daughter-cell separation) took ~29.2 min, ~7.4 min, ~37.7 min, ~24.0 min, and >72.5 min at 25°C (only quantified the separated cells by the end of the movie so the septum maturation time was underestimated), respectively. All these processes showed significant delay compared to WT cells that took ~24.7 min, ~5.1 min, ~27.2 min, ~10.1 min, and ~16.2 min (p < 0.01), respectively (Figure 3, A and B). Thus, Pmo25 plays important roles in cytokinesis.

When grown longer at 36°C, unlike in WT, the contractile ring became unstable, frayed, or disintegrated during or before its constriction in *pmo25-2* mutant (Figure 3C). Based on these results, we hypothesized that Pmo25 may also affect the contractile ring integrity or stability. To test this hypothesis further, we examined the ring stability in the (1,3)β-glucan synthase mutant *bgs1/cps1-191*, which significantly delays contractile-ring constriction with a super stable ring ^74,75,85^. As expected, the percentage of *bgs1-191* cells with rings reduced dramatically by the *pmo25* mutation. After shifting to 36°C for 2 h, ~45% of *bgs1-191* cells had a contractile ring, whereas only ~5% of *bgs1-191 pmo25-2* cells had a ring (Figure 3, D and E). We reasoned that the lack of the ring in *bgs1-191 pmo25-2* cells may be caused by the faster constriction and/or ring collapse. In 70-min time lapse movies at 36°C, essentially all the rings in *bgs1-191* cells remained stable and did not constrict obviously. In contrast, ~55% of the rings in *bgs1-191 pmo25-2* cells were disrupted or collapsed during the movie (Figure 3, D and E, movies 1 and 2). These results suggested that Pmo25 is important for contractile-ring stability/integrity during cytokinesis.

**Figure 4.**
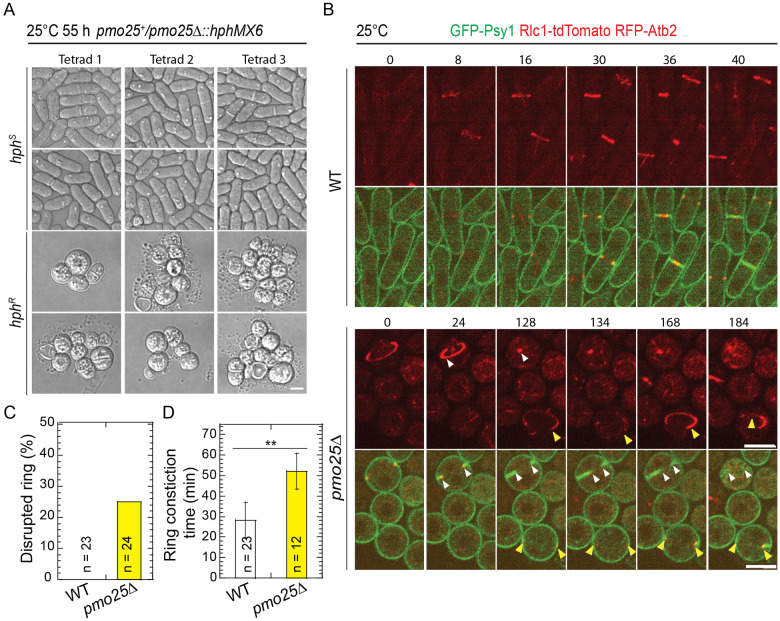
Pmo25 is important for cell polarization and contractile ring stability. (A) Morphology of *pmo25Δ* mutant. One copy of *pmo25* was deleted in diploid cells. After tetrad dissection, spores were grown at 25°C for ~55 h, then imaged under microscope and tested for hygromycin resistance (*hph^R^*) or sensitivity (*hph^S^*). (B) Time courses (in min) of GFP-Psy1 Rlc1-tdTomato RFP-Atb2 in WT and *pmo25Δ* cells at 25°C. Time 0 is the start of the movies. Upper panel, Rlc1 and Atb2 signals; Lower panel, merge. Arrowheads mark examples of disrupted rings. Bars, 5 μm. (C) Percentage of cells with contractile ring break-down before or during constriction in movies (≥3 hrs) as in (B). (D) Times of the contractile ring constriction in WT and *pmo25Δ* cells measured from movies as in (B). **p < 0.001.

Temperature-sensitive *pmo25* mutants may still retain some Pmo25 functions even at 36°C. To confirm Pmo25’s role in contractile-ring stability, we examined cytokinesis in *pmo25*Δ cells grown at 25°C using Tetrad Fluorescence Microscopy ^86-88^. As reported ^33,34^, *pmo25*Δ cells became rounder, ceased growth, and lysed after several divisions, in contrast to the rod-shaped WT cells (*hphS*) after tetrad dissection of *pmo25*^+^/*pmo25*Δ cells (Figure 4A). Similar to the *pmo25* temperature-sensitive mutants, *pmo25*Δ cells exhibited a delay in cytokinesis. Except for the ring sliding from the cell center, ~25% of contractile rings in *pmo25*Δ, but not in WT cells, collapsed during formation, maturation, or constriction (Figure 4, B and C, movies 3 and 4). Although the ring constriction took longer in *pmo25*Δ cells, ~52.3 min compared to ~27.8 min in WT cells (Figure 4D; p < 0.001), the constriction rate showed no obvious difference, ~0.39 μm/min in *pmo25*Δ cells compared to ~0.41 μm/min in WT cells (p = 0.51). These results confirm that Pmo25 is required for contractile-ring stability and integrity during cytokinesis.

### Pmo25 binds Cdc4 and regulates Cdc4 ring stability

**Figure 5.**
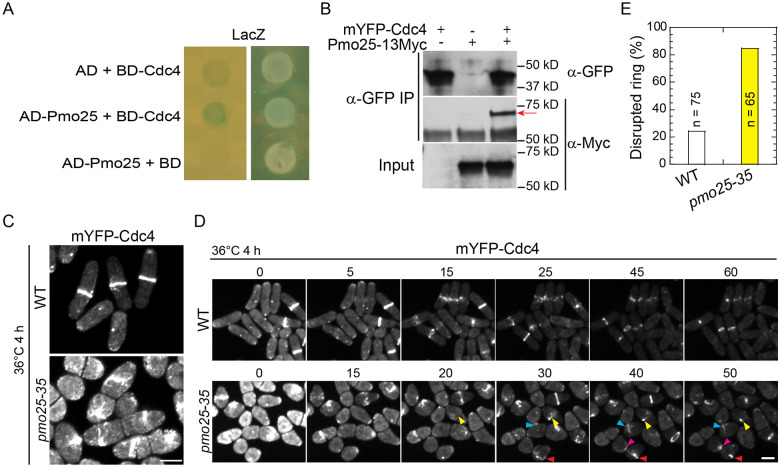
Pmo25 interacts with myosin-II essential light chain Cdc4. (A) Pmo25 and Cdc4 interact in yeast two-hybrid assays. *S. cerevisiae* MaV203 cells were transformed with AD-Pmo25 and/or BD-Cdc4 with LacZ as a reporter. (B) Co-IP of Pmo25 and Cdc4. Extracts of cells expressing Pmo25-13Myc and/or mYFP-Cdc4 (JW910, JW8662 and JW9877) were immunoprecipitated with anti-GFP polyclonal antibody, then detected by anti-Myc or anti-GFP monoclonal antibodies, respectively. (C-E) *pmo25* affects mYFP-Cdc4 ring stability. Single image (C) and selected images from time lapse (D, in min) of mYFP-Cdc4 in WT (JW10097) and *pmo25-35* mutant (JW10098) grown at 36°C for 4h before imaging at 36°C. Arrowheads of the same color mark the same cell. (E) Quantification of cells with mYFP-Cdc4 ring break-down before or during ring constriction in movies as in (D). Bars, 5 μm.

Cells lacking Pmo25 lead to contractile-ring instability during its formation or constriction, suggesting that Pmo25 may interact with other proteins in the contractile ring. To test this idea, we first tested if Pmo25 interacts with the main contractile ring components such as the IQGAP scaffold protein Rng2, formin Cdc12, F-BAR protein Cdc15, myosin II heavy chain Myo2 and essential light chain Cdc4 by yeast-two-hybrid assays. Although not very strong, a positive interaction between Pmo25 and Cdc4 was detected (Figure 5A). To address whether Pmo25 binds Cdc4 in fission yeast cells, we performed Co-IP as in Figure 1 and found that Pmo25-13Myc co-immunoprecipitated with mYFP-Cdc4 (Figure 5B).

To dissect the contribution of Pmo25 to Cdc4 function, we examined Cdc4 localization in *pmo25* mutants. Neither COOH- nor NH_2_-tagged Cdc4 is fully functional; therefore, we used mYFP-Cdc4, the more functional one, to observe Cdc4 localization in *pmo25* mutant. After shifting the cells to 36°C for 4 h, ~20% of mYFP-Cdc4 rings collapsed in *pmo25*^+^ cells, in contrast, > 80% of mYFP-Cdc4 rings collapsed in *pmo25-35* cells (Figure 5, C-E). In addition, we observed the synthetic genetic interactions between *pmo25* mutants and other contractile ring mutants, such as *cdc12*, *rng2*, *myo2*, *cdc4*, and *rlc1* mutants. Incomplete and disorganized septa were often observed in the double mutants. These results indicate that Pmo25 associates with Cdc4 to regulate contractile-ring stability during cytokinesis.

### Pmo25 plays a role in Sid2/Mob1 recruitment or maintenance at the division site

**Figure 6.**
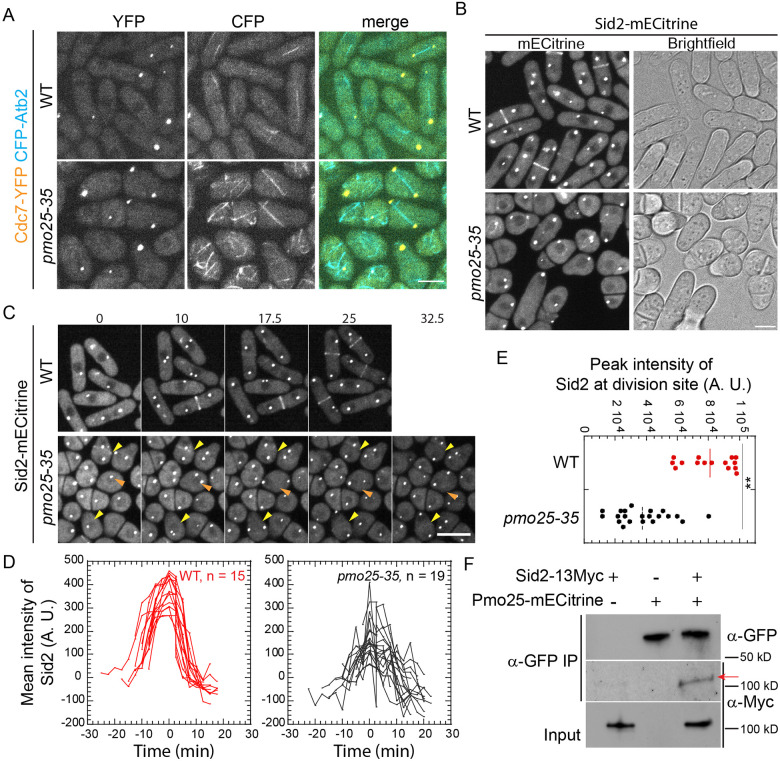
Pmo25 binds the SIN kinase Sid2 and is involved in Sid2’s recruitment to the division site. (A and B) Localization of the SIN kinases Cdc7 (A) and Sid2 (B) in *pmo25-35* mutant. Cells of WT (JW10070 and JW10139) and *pmo25-35* mutant (JW10063 and JW10138) expressing Cdc7-YFP or Sid2-mECitrine were grown at 25°C for ~36 h, then shifted to 36°C for 4 to 5 h before imaging at 36°C. (C-E) Time course in minutes (C), mean intensity (D), and peak intensity (E) of Sid2-mECitrine at the division site. Cells (JW10138 and JW10139) were grown at 25°C for ~36 h, then shifted to 36°C for 5 h before imaging at 36°C. In (C), yellow arrowheads: cells with weak Sid2 signal at the division site; orange arrowheads: almost no Sid2 signal at the division site during cytokinesis. (D) Time zero marks Sid2 reaches peak intensity at the division site. (E) **p<0.0001. (F) Co-IP of Pmo25 with Sid2. Extracts of cells expressing Pmo25-mECitrine and/or Sid2-13Myc (JW1369, 7943 and 10082) were immunoprecipitated with anti-GFP polyclonal antibody, then detected by anti-Myc or anti-GFP monoclonal antibodies, respectively. Bars, 5 μm.

Pmo25 mediates the signaling linkage between SIN and the network for cell morphogenesis/separation following cytokinesis ^33,53^. Pmo25 localization at the SPBs is controlled by Cdc7 and Sid1 kinases but not Sid2 kinase in the SIN pathway ^33,34^. Inactivation of SIN signaling leads to a failure of contractile-ring maintenance and septum formation, and it ultimately leads to cell lysis during cytokinesis due to the defective septum ^8,12,24,89^. The cytokinetic defects observed in *pmo25* mutants promoted us to investigate if Pmo25 may feedback to the SIN signaling. We first tested Cdc7 localization in *pmo25* mutant cells 4 h after shifting cells to 36°C. In WT cells, consistent with previous reports ^90-93^, Cdc7-YFP localized to the two SPBs at early mitosis, then associated and increased recruitment to one SPB during anaphase B (Figure 6A). Similarly, *pmo25-35* cells showed Cdc7-YFP localized to the two SPBs at early mitosis, and one signal-increased SPB during anaphase B (Figure 6A).

Surprisingly, the division-site localization of Sid2, the downstream kinase of the SIN pathway, was significantly affected in *pmo25* mutant cells (Figure 6, B-E, movies 5 and 6). In WT cells, 5 h after shifting to 36°C, Sid2-mECitrine was still recruited to the division site during the late cytokinesis as reported (Figure 6, B and C, movie 5). In contrast, in *pmo25-35* cells at 36°C, the division site recruitment of Sid2-mECitrine was significantly decreased (Figure 6, B and C, movie 6). We also found that some round *pmo25-35* cells, which may indicate more severe Pmo25 activity defect, showed no detectable Sid2 signal at the division site (Figure 6, B and C, movie 6). To further confirm the effect of Pmo25 on Sid2 distribution at the division site, we measured Sid2-mECitrine intensity at the division site in time-lapse movies. In WT cells, recruitment of Sid2 to the division site began before spindle break-down (indicated by Sid2 SPBs movement), and these cells rapidly reached the peak intensity. However, in *pmo25-35* cells, the division site recruitment of Sid2 was significantly compromised, the peak Sid2 intensity decreased ~50% in *pmo25-35* than WT cells (Figure 6, D and E). Consistently, the Sid2 binding partner Mob1 showed similar defects in division site recruitment in *pmo25-35* cells (Figure S1, A and B). The dependence of Sid2 division-site localization on Pmo25 suggest they may interact physically with each other. Indeed, after immunoprecipitating Pmo25 with anti-GFP antibody, Sid2 was detected in the cells co-expressing Pmo25-mECitrine and Sid2-13Myc (Figure 6F). Taken together, these results suggest that Pmo25 associates with Sid2 kinase and plays a role in the recruitment of the Sid2/Mob1 complex to the division site during cytokinesis.

**Figure 7.**
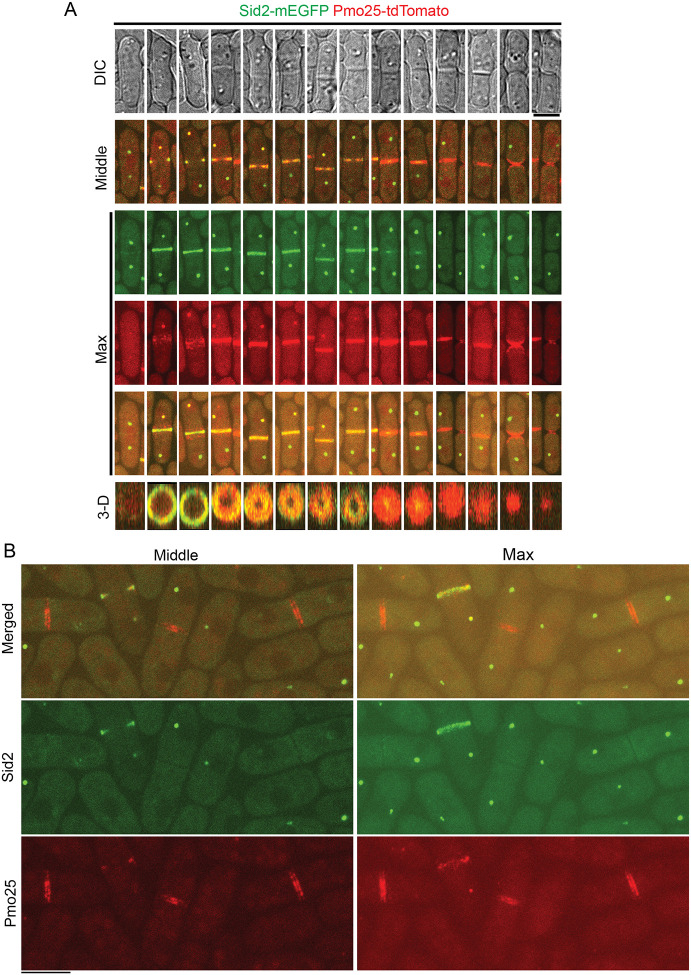
Colocalization of Sid2 and Pmo25 at the SPB and division site during cytokinesis. (A) Colocalization of Sid2 and Pmo25 at the SPB and the division site. Single time-point images of cells expressing both Sid2-mEGFP and Pmo25-tdTomato (JW10302) are ordered chronologically based on septum morphology and Sid2 and Pmo25 signals. DIC, middle focal plane, maximal intensity projection (19 slices with 0.3 μm spacing), and 3-D projections are shown. Cells were grown at 25°C for ~48 h before imaging. (B) SoRa imaging of Pmo25 and Sid2 at the division site in the middle focal plane and max projections. Cells expressing Sid2-mEGFP and Pmo25-tdTomato (JW10302) were grown at 25°C for ~48 h before imaging. Bars, 5 μm.

**Figure 8.**
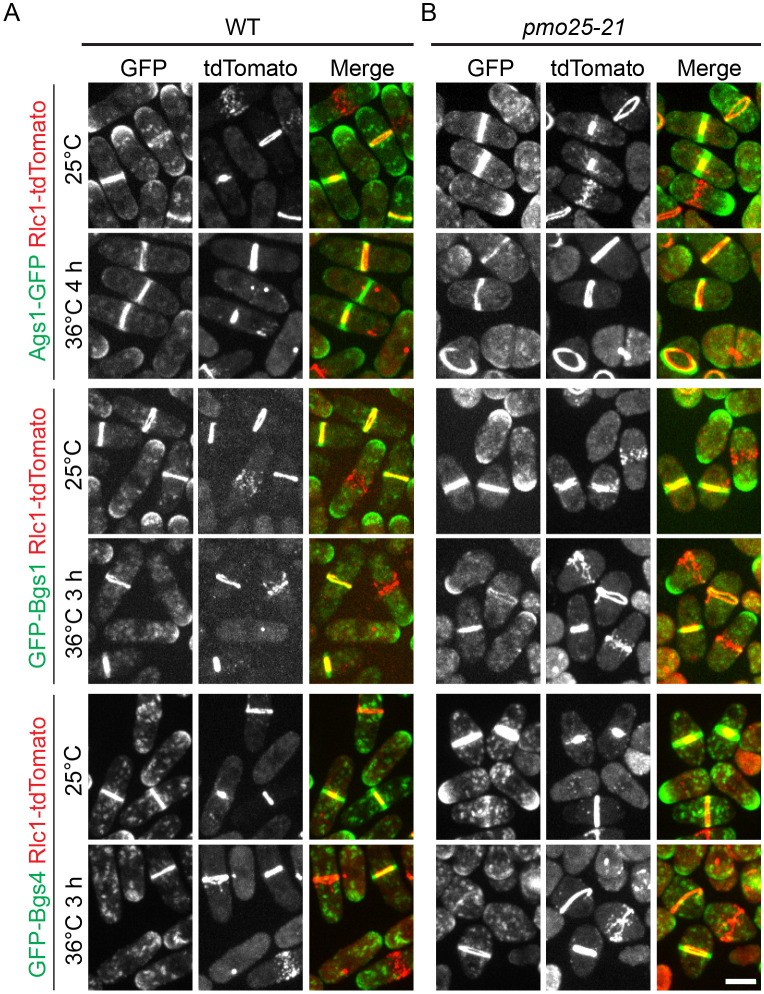
Localization of glucan synthases in *pmo25-21* mutant. (A and B) Localization of Bgs1, Bgs4, and Ags1 in (A) WT (JW5429, JW6153, JW6810) and (B) *pmo25-21* mutant (JW8567, JW8574, JW8677). Rlc1-tdTomato marks the contractile ring. Cells were grown at 25°C for ~36 h then imaged, or shifted to 36°C for 3 h or 4 h before imaging at 36°C. Bar, 5 μm.

The roles of Pmo25 in Sid2’s recruitment and/or maintenance at the division site and their physical interactions in co-IP suggest that they must overlap temporally and spatially in *S. pombe* cells, which had not been examined before. Therefore, we tested their spatiotemporal relationship using confocal imaging, time-lapse, and SoRa high spatial resolution imaging in cells expressing both Sid2-mEGFP and Pmo25-tdTomato (Figures 7 and S2; and Movie 7). As reported before, Sid2 localizes to one SPB during interphase and then both SPBs and the division site during mitosis and cytokinesis ^26,27,94-98^. Pmo25 localizes to both SPBs transiently during early anaphase, then only to the new SPB with active Cdc7 kinase ^33,34^. Sid2 and Pmo25 colocalized in the new SPB from mid to late anaphase until Pmo25 disappeared from the SPB near the end of contractile ring constriction in cells with a closed septum (Figures 7A and S2, and Movie 7). Sid2 was observed to form a discrete ring at the division site several minutes before Pmo25 during late anaphase (Figures 7A and S2; and Movie 7). It is interesting that Pmo25 appeared as a diffuse band on both sides of Sid2 at the division site first and then coalesced into a discrete ring that colocalized with Sid2 (Figures 7A and S2; and Movie 7). Sid2 and Pmo25 spread to the whole division plane along with contractile-ring constriction and septum formation to form a washer-like structure and then a disc (Figure 7A). Sid2 faded away from the division site several minutes after septum closure and at least 10 minutes after Pmo25 disappeared from the new SPB (Figure S2). Pmo25 stayed at the division site until the daughter-cell separation (Figure 8A). High-resolution SoRa microscopy confirmed that Pmo25 formed double discs ^33,34^, which correspond to the two new layers of the plasma membrane at the division site (Figure 8B). These results were confirmed using cells expressing both Sid2-mEGFP and Pmo25-mScarlet-I. Collectively, these data indicate that Sid2 and Pmo25 colocalize at the new SPB and the plasma membrane at the division site for >30 minutes and support their physical and functional interactions.

### Pmo25 is involved in recruitment of glucanase Eng1 to the division site

We next investigated the function of the Pmo25 and Ync13 interaction. Ync13 affects septum integrity by regulating the distribution of glucan synthases and glucanases at the division site ^58^. Our data show that cytokinesis and cell separation are impaired in *pmo25* mutants; therefore, we examined whether *pmo25* mutants affect glucan synthases and/or glucanases. First, we tested the localizations of the glucan synthases Bgs1, Bgs4, and Ags1, which assemble a three-layer septum composed of mainly α- and β-glucan during cytokinesis ^67-69^. Rlc1-tdTomato was used as the contractile-ring marker. As reported, GFP-Bgs1, GFP-Bgs4, and Ags1-GFP are transported by secretory vesicles and concentrated to the plasma membrane at the growing cell tips during interphase and recruited to the division site during the contractile ring maturation and constriction and, then remain at the division site until daughter-cell separation (Figure 8A). In *pmo25-21* mutant cells, which also rescued *ync13Δ* (data not shown), all three glucan synthases still localized to vesicles, cell tips, and the division site at comparable levels to WT cells at both 25°C and 36°C (Figure 8B).

**Figure 9.**
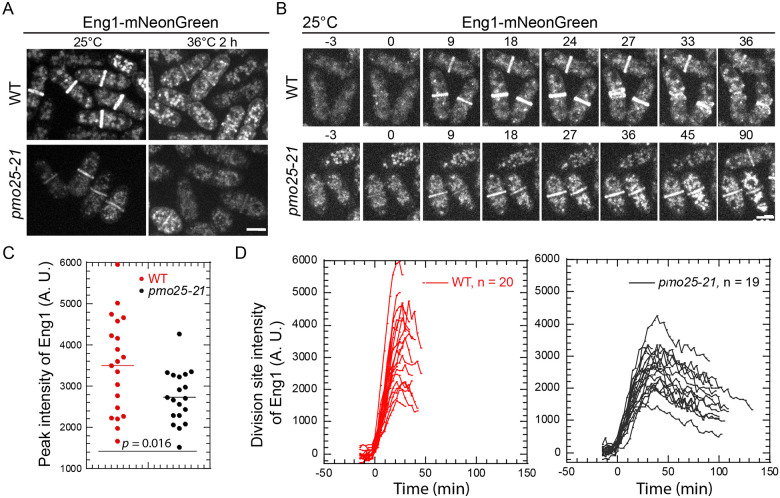
*pmo25-21* mutant affects β-glucanase Eng1’s recruitment to the division site. (A) Localization of Eng1 in WT (JW8489) and *pmo25-21* mutant (JW8488). Cells were grown at 25°C for ~36 h then imaged, or shifted to 36°C for 2 h before imaging at ~36°C. (B) Selected micrographs of Eng1 time course (in min) in WT (JW8489) and *pmo25-21* mutant (JW8488) grown at 25°C for ~36 h before imaging. Time 0 marks Eng1 appearance at the division site. (C and D) Eng1 peak intensity (C) and time course of its mean intensity (D) at the division site in WT (JW8489) and *pmo25-21* cells (JW8488) grown at 25°C as in (B). Time 0 marks Eng1 appearance at the division site. Bars, 5 μm.

Next, we examined the distribution and intensity of the endo-1,3-β-glucanase Eng1 at the division site, which is involved in primary septum digestion to separate the two daughter cells ^76,77,99-101^. Eng1 was concentrated to the division site during late cytokinesis in WT cells as reported (Figure 9, A and B). However, in *pmo25-21* cells, the division site level of Eng1 was significantly decreased even at 25°C. Because the division site signals of Eng1-mNeonGreen were weak in WT cells after shifting to 36°C (Figure 9, A and B), we measured Eng1-NeonGreen intensities during time lapse movies at 25°C (Figure 9, C and D). In *pmo25-21* cells, the peak levels of Eng1-NeonGreen at the division site were significantly lower than that in WT cells, and the maximal recruitment of Eng1-NeonGreen to the division site also took longer in *pmo25-21* mutant cells (Figure 9, C and D). Eng1 also stayed longer at the division site (Figure 9D), maybe due to the cytokinesis delay in *pmo25-21* cells. These data indicate that Pmo25 plays a role in the glucanase Eng1 recruitment to the division site, which contributes to the delay of daughter-cell separation in *pmo25-21* mutants.

## DISCUSSION

In this study, we identified and confirmed that the MO25 protein Pmo25 is a binding partner of the Munc13/UNC-13 protein Ync13 through yeast two-hybrid screen, Co-IP, and in vitro binding assays. Here we found that Pmo25 plays important roles in contractile-ring stability, SIN signaling, and daughter-cell separation during cytokinesis. Because both MO25 and Munc13/UNC-13 proteins are highly conserved during evolution ^4,37,38,42,43,45,46,52,54,58,65^, our data provide insights into their potential interactions and functions in cell morphogenesis and cell division in other systems.

### Pmo25 controls contractile-ring stability and regulates the SIN signaling during cytokinesis

Previous studies found that Pmo25 is a component of the MOR network and is essential for Orb6 activation during cell polarization and morphogenesis ^33,34,53^. Pmo25 localizes to one or both SPBs during mitosis and to the division site during cytokinesis ^33,34,53^. It mediates signaling connection between the MOR and SIN pathways ^33,34,53^. It was reported that *pmo25* mutants are defective in cell separation after septum formation with an increased septation index and some multiseptated cells ^33,34^. However, the molecular mechanism of how Pmo25 regulates cytokinesis remains a mystery. In budding yeast, the MO25 protein Hym1 has been shown to be involved in cell separation but not in contractile-ring stability yet ^4,47,102^.

Here we presented several lines of evidence that Pmo25 is essential for contractile-ring stability and maintenance. First, the contractile ring frequently collapses, frays, or disintegrates before or during constriction in *pmo25* temperature-sensitive or deletion mutants, or in combination with the *bgs1/cps1-191* mutant. Second, Pmo25 physically interacts with Cdc4 and most mYFP-Cdc4 rings collapse during cytokinesis. Cdc4 is an essential protein in contractile-ring stability and maintenance as a binding partner of myosin-IIs Myo2 and Myp2, myosin-Vs, and IQGAP Rng2 ^82,103-107^. Third, *pmo25* mutants have strong genetic interactions with mutations affecting ring stability and maintenance such as mutations in *cdc12*, *rng2*, *myo2*, *cdc4*, and *rlc1*. Thus, Pmo25 plays important roles in contractile-ring stability before and during its constriction.

Sid2 is the most downstream kinase in the SIN pathway. It localizes to the SPBs and the division site ^26,27,94^. It is known that the SIN pathway regulates Pmo25 localization to the SPBs ^33,34^. In this study, we showed that Pmo25 also regulates Sid2-Mob1 localization at the division site. We found that the levels of Sid2 and its binding partner Mob1 at the division site are dramatically reduced in *pmo25* mutant cells. Their peak intensity and kinetics of recruitment at the division site are both severely compromised. In addition, Sid2 and Pmo25 colocalize at the division site and the SPBs during cytokinesis, and they interact in Co-IP assays, which has not been detected before. It will be interesting to test if MO25 interacts with the tumor suppressor NDR kinase STK38, the Sid2 homolog in mammalian cells ^108-112^. The SIN pathway is known to regulate contractile-ring stability and maintenance ^8,12,24,89^, so it is possible that Pmo25 is one of the players that regulate the contractile ring during cytokinesis through the SIN pathway. MO25 forms a heterotrimeric complex with the pseudokinase STE20-related adaptor (STRAD) and the tumor suppressor liver kinase 1 (LKB1). The LKB1-STRAD-MO25 complex stabilizes a closed conformation of STRAD and triggers LKB1 nucleocytoplasmic shuttling and activation ^36-39^. It seems that Pmo25 also forms a heterotrimeric complex with the Sid2-Mob1 kinase complex during cytokinesis. Thus, other MO25 proteins may also interact with NDR kinases in the Hippo pathways to regulate cytokinesis.

### Pmo25 interacts with Ync13 to regulate the exocytosis of the glucanase Eng1

We showed that Pmo25 and Ync13 physically interact with each other by yeast two-hybrid, Co-IP, and in vitro binding assays. Their interaction is direct with a relatively strong affinity (K_d_ < 1 μM). The Pmo25 level at the division site is not affected by *ync13Δ*. By contrast, Ync13 fluorescence intensity at the division site increases by almost 50% in *pmo25* mutant cells. Further studies are needed to elucidate why the Ync13 level at the division site increases in *pmo25* mutant cells. What are the functions of the Ync13-Pmo25 interaction in fission yeast? Ync13 and Pmo25 partially colocalize on the plasma membrane at the division site with Ync13 being more concentrated at the leading edge. Because *ync13* mutants have no effects on contractile-ring stability and maintenance and Pmo25 level and dynamics at the division site are normal without Ync13,the Ync13-Pmo25 interaction is less likely to be involved in regulating the contractile ring. It is more likely the interaction is important for recruiting the glucanase Eng1 for daughter-cell separation via exocytosis. The Eng1 level and distribution at the division site are compromised in both *ync13* and *pmo25* mutants ^58^. Thus, both Ync13 and Pmo25 play important roles in septum formation and daughter cell separation.

Our data suggest several novel functions of Pmo25 that modulate several cellular processes such as exocytic trafficking, contractile ring stability and constriction, and cell separation. Pmo25 is involved in several modules during cytokinesis, which is consistent with the fact that MO25 serves as master regulators in other organisms. How these same spatial modules coordinate cell events, and whether Pmo25 regulates them in different ways need further investigation.

### Limitations of the study

The dynamics of Ync13 in *pmo25* mutants needs to be tested using FRAP assays, which is challenging because Ync13 signal is weak and Ync13 is highly dynamic at the division site with half times of 2 to 3 seconds in FRAP assays ^58^. Pmo25 and Ync13 interact with each other and partially colocalize at the division site. It is unknown why Pmo25 (19-329) but not full length Pmo25 interacts with Ync13. The first 18 residues of Pmo25 are upstream of the armadillo-like helical repeat and have no obvious motif. Future studies are needed to figure out the functions of the NH2 terminal part of Pmo25 and those of the armadillo-like repeats. Pmo25 but not Ync13 also localizes to the SPBs. Although both *ync13* and *pmo25* mutants are defective in cytokinesis, their morphologies are not identical except both mutant cells lyse. *pmo25* but not *ync13* mutant cells also lose polarity. Thus, Ync13 and Pmo25 must also have other binding partners and independent functions besides working together, which need to be examined in future studies. Pmo25 also interacts with Cdc4 and Sid2 directly or indirectly, which have not been tested by in vitro binding assays. More studies are needed to determine if Pmo25 regulates the contractile-ring stability through proteins other than Cdc4 and Sid2. Pmo25 affects the Sid2-Mob1 complex recruitment to the division site, and this might be independent from the MOR pathway, as *orb6-25* mutant did not show any strong defects in the division site recruitment of the Sid2-Mob1 complex as in *pmo25* mutants, although we cannot rule out the possibility of the remaining MOR function in *orb6-25* ^31,32,53^. Domain analyses in future studies are needed to dissect how each domain contributes to the interactions between Pmo25 and different proteins, and whether Pmo25 also controls Sid2 kinase activity. Sid2 kinase has several substrates in the contractile ring such as the formin Cdc12, anillin Mid1, phosphatase Cdc14. It will be interesting to explore their functional relationship with Pmo2

## STAR*METHODS

### EXPERIMENTAL MODEL AND SUBJECT DETAILS

#### Fission Yeast

Yeast strains used in this study are listed in Supplemental Table S1. Cells were woken up from −80°C stocks and grown on YE5S plates at 25°C for ~2 d, and then fresh cells were inoculated into YE5S liquid medium and grown at 25°C for ~36 h at log phase (diluted twice daily) before imaging except where noted. Detailed growth conditions for individual experiments are in figure legends.

#### Escherichia coli

The plasmids were transformed into BL21 (DE3) pLysS cells (Novagen, 694513) for protein expression. MBP-Ync13-full length-6His and MBP-Ync13-(591-1130)-6His were induced with 0.2 mM isopropyl-β-D-thiogalactoside (IPTG) at 17°C for 36–48 h. Pmo25-full-length-6His and Pmo25-(19–329)-6His were expressed with 0.5 mM IPTG at 25°C for 15 h.

### METHOD DETAILS

#### Strains, genetics, and cellular methods

Strains used in this study are listed in Supplemental Table S1. PCR-based gene targeting and genetic crosses were performed using standard methods ^115,116^. All tagged genes are expressed under endogenous promoters and integrated into the native chromosomal loci except *GFP-psy1*, which is at *leu1* locus. *pmo25* deletion mutant was constructed in diploid strain *h*^+^*/h*^−^ *rlc1-tdTomato-natMX6/rlc1-tdTomato-natMX6 leu1*^+^*::GFP-psy1/ leu1*^+^*::GFP-psy1 Patb2-mRFP-atb2/Patb2-mRFP-atb2 ade6-M210/ade6-M216 ura4/ura4* using the *hphMX6* marker.

#### Yeast two hybrid screen and assays

Yeast two hybrid screen was carried out as previously described ^117^. The fragments corresponding to Ync13 full length (1237 aa), NH2-terminal (1-590 aa), and COOH-terminal (591-1237 aa) were amplified from a cDNA library and cloned into BD (DNA binding domain) pGBT9 vector, and co-expressed in *S. cerevisiae* MaV203 strain with prey library pACT2 (lab stock) by sequential transformation. The selection was performed with reporter genes: *URA3*^+^, *HIS3*^+^, and *LacZ*. For X-gal overlay assay, fresh colonies from leucine and tryptophan selection plate were re-streaked on YPD (yeast extract-peptone-dextrose) plates and grew overnight. ~8 ml chloroform per plate was used to permeabilize cells for 10 min and then dried for additional 2 min before overlay. The overlay solution was prepared in 25 ml PBS (pH 7.5) with 0.5% agarose and 500 μl X-gal (20 mg/ml stock in DMSO). The overlaid plates were incubated at 30°C and checked for development of blue color every 30 min. Plasmid DNAs from the positive colonies were isolated and sequenced.

To test the interaction between Pmo25 and the contractile ring components, the full length cDNA of Pmo25 was cloned into VP16 transcription activation domain (AD) vector, pVP16, and co-transformed into *S. cerevisiae* MaV203 strain with pGBT9 vector expressing full length Rng2, Cdc15, Cdc12, Myo2, and Cdc4 ^82,118,119^, respectively. The selection was performed with reporter genes as described above. All constructed plasmids were confirmed by Sanger sequencing.

#### IP and Western blotting

Co-IP and Western blotting were performed as previously described ^82,120,121^. Yeast cells expressing tagged proteins under the control of native promoters were harvested at exponential phase, washed twice with ddH2O, frozen immediately with liquid nitrogen, and then lyophilized. Lyophilized cells were further ground into a homogeneous powder in mortar with liquid nitrogen. ~100 mg cell powder for each sample was dissolved in IP buffer (50 mM 4-(2-hydroxyethyl)-1-piperazineethanesulfonic acid [HEPES], pH 7.5, 150 mM NaCl, 1 mM EDTA, 0.5% NP-40 [for Ync13 and Pmo25 IP, 0.5% Triton and 0.5% CHAPS were used], 0.1 mM Na3VO4, 1 mM PMSF, and EDTA-free protease inhibitor cocktail [cOmplete, Roche]) on ice, then centrifuged at 10,000 g for 20 min at 4°C. 30 μl of protein G covalently coupled magnetic Dynabeads (100.04D; Invitrogen, Carlsbad, CA) per sample was washed three times with cold phosphate-buffered saline (PBS) buffer (2.7 mM KCl, 137 mM NaCl, 10 mM Na2HPO4, and 2 mM KH2PO4, pH 7.4), and 5 μl of rabbit anti-GFP antibody (NB600-308; Novus Biologicals, Littleton, CO) per sample in PBS buffer was added to beads. After incubation for 1 h at room temperature, the beads were washed three times with PBS buffer and twice with the IP buffer. 300 μl cell extracts from centrifuged supernatants were mixed with antibody-coupled Dynabeads and incubated for 1.5 h at 4°C. The precipitated beads were washed twice with wash buffer I (50 mM HEPES, pH 7.5, 150 mM NaCl, 1 mM EDTA, 0.1% NP-40) and three times with wash buffer II (50 mM HEPES, pH 7.5, 150 mM NaCl, 1 mM EDTA), then dissolved in sample buffer and boiled for 5 min. After separation of proteins in SDS–PAGE, proteins were detected by Western blotting using monoclonal anti–GFP/YFP antibody (1:2500 dilution; Roche) or monoclonal anti-Myc antibody (9E10, 1:1000 dilution; Santa Cruz Biotechnology, Santa Cruz, CA).

#### Protein purification and in vitro binding assays

The MBP-TEV-GGSGGS fragment was first inserted into the pET21a vector upstream of the *Bam*HI site using Gibson assembly ^122^ to generate the pET21a-MBP construct. The full-length Ync13 cDNA was subsequently cloned into the pET21a-MBP vector between the GGSGGS linker and the C-terminal 6×His tag via Gibson assembly. Similarly, full-length Pmo25 and its truncated form (residues 19–329) were cloned into the pET21a vector between the N-terminal T7 tag and the C-terminal 6×His tag using the same method. All constructs were verified by DNA sequencing.

MBP-Ync13-full length-6His, MBP-Ync13-(591-1130)-6His, Pmo25-full length-6His, and Pmo25-(19-329)-6His were purified with Talon metal affinity resin (635501; Clontech, Mountain View, CA) using extraction buffer (50 mM sodium phosphate, pH 8.0, 450 mM NaCl, 10 mM β-mercaptoethanol, 1 mM PMSF, and 10 mM imidazole) with EDTA-free protease inhibitor tablet (Roche) as described before ^123^. Proteins were eluted with elution buffer (50 mM sodium phosphate, pH 8.0, 450 mM NaCl, 10 mM β-mercaptoethanol, 1 mM PMSF, and 200 mM imidazole). The purified proteins were then dialyzed into the binding buffer (137 mM NaCl, 2 mM KCl, 10 mM Na_2_HPO_4_, 2 mM KH_2_PO_4_, 0.5 mM dithiothreitol, and 10% glycerol, pH 7.4).

For in vitro binding assays between Ync13 and Pmo25, we incubated MBP-Ync13-full length-6His, MBP-Ync13-(591-1130)-6His or MBP-6His control with 500 μl amylose beads for 1 h at 4°C and then washed the beads eight times with 1 ml of the binding buffer each time to remove unbound proteins. Then Pmo25-full length-6His or Pmo25-(19-329)-6His was incubated with the 100 μl beads with bound MBP-Ync13 proteins for 1 h at 4°C. After 4 washes with 1 ml of the binding buffer each time, the beads were boiled with sample buffer for 5 min. Then the samples were run on SDS–PAGE gel and detected with Coomassie Blue staining.

To measure the Kd between Ync13-(full length/591-1130) and Pmo25-(19–329), we followed the described methods and guidelines ^123,124^. Various concentrations of MBP-Ync13-(full length/591-1130)-6His immobilized on amylose beads were incubated with a fixed low concentration of Pmo25-(19–329)-6His for 1h at 4°C. After incubation, the beads were spun down at 1,000 g for 1 min, supernatant samples removed from the reactions were boiled with sample buffer, run on an SDS-PAGE gel, and detected with Coomassie Blue staining. Total amount of bead bound Pmo25-(19-329)-6His was calculated by subtracting the remaining proteins in the supernatant from the total input. The Kd was calculated using one site binding Equation in Graphpad Prism 9.5.0.

#### Microscopy and data analyses

For microscopy imaging, cells were woken up from −80°C stocks and grown on YE5S plates at 25°C for ~2 d, and then fresh cells were inoculated into YE5S liquid medium and grown at 25°C for ~36 h at log phase (diluted twice daily) before imaging except where noted. Microscopy sample preparations were carried out as described ^80,87,120,125^. Briefly, cultured cells were collected by centrifugation at 3,000 to 3,500 rpm, washed once with EMM5S, and then washed with EMM5S containing 5 μM *n*-propyl-gallate to reduce autofluorescence and protect cells from free radicals during microscopy. For imaging *pmo25Δ* using fluorescence confocal microscope, dissected tetrads were grown on YE5S agar plate for ~16 h, then a fraction of WT cells and all *pmo25Δ* cells (predicted by phenotype) were collected to separate positions by using glass needle on the tetrad dissection microscope, grew further on YE5S agar plate at 25°C. The agar piece with cells was cut out and placed face down on a 35-mm dish with a glass coverslip bottom, an 18 x 18 mm coverslip was covered on the top as well to slow down the drying of agar media and imaged directly. Hygromycin sensitivity was checked with the leftover WT cells.

To observe *pmo25* deletion mutant phenotype under DIC in Figure 4A, the YE5S agar plate with dissected tetrads was cut out and placed on a glass slide, after covered with a glass coverslip, sample was sealed with VALAP and imaged with a 100×/1.4 numerical aperture (NA) Plan-Apo objective lens on a Nikon Eclipse Ti inverted microscope (Nikon, Melville, NY) equipped with a Nikon cooled digital camera DS-QI1. All fluorescence microscopy was carried out at ~23°C on a PerkinElmer spinning disk confocal system (UltraVIEW Vox CSUX1 system; PerkinElmer, Waltham, MA) on a Nikon Ti-E microscope with Hamamatsu EMCCD camera C9100-23B and Plan-Apo 100x/1.45 NA objective; or on a Nikon CSU-W1 SoRa spinning disk confocal microscope with Hamamatsu ORCA Quest qCMOS camera C15550 on Nikon Eclipse Ti2 microscope and Apo TIRF 100x/1.49 NA oil, Plan Apo λD 100x/1.45 NA oil, or Plan Flour 100x/1.30 NA oil objectives.

Images and data were collected and analyzed by Volocity, NIS Elements, and Fiji software. The fluorescence intensity at the division site was measured in the images that were projected with sum intensity of 0.5 μm-spaced Z-slices as described previously ^126,127^. ROI (region of interest) covering the signal at the division site was drawn to measure the mean intensity, and ~2× ROI was used to measure and calculate background intensity ^58,118,120^. Micrographs shown in the figures are maximum projections except where noted. Statistical tests were performed using a two-tailed Student’s *t* test.

## Supplementary Material

Supplement 1

## Figures and Tables

**Table:** KEY RESOURCE TABLE

REAGENT or RESOURCE	SOURCE	IDENTIFIER
Antibodies		
Rabbit anti-GFP	Novus Biologicals	NB600-308
Anti-GFP/YFP monoclonal	Roche	11814460001
Anti-Myc	Santa Cruz Biotechnology	9E10
Anti-mouse IgG	Sigma	A4416
Chemicals and reagents		
Dynabeads	invitrogen	10004D
BL21(DE3)pLysS	Novagen	694513
Protease inhibitor cocktail cOmplete	Roche	11873580001
Amylose beads	New England Biolabs	8021S
n-propyl-gallate	Thermo Fisher Scientific	P3130-100G
X-Gal	Thermo Fisher Scientific	BP1615-1
Gelatin	Sigma-Aldrich	G2500-500G
BioMax MR film	Kodak	Z350370-50EA
pACT2 yeast two hybrid library	Lab stock	N/A
Talon metal affinity resin	Clontech	635501
SuperSignal west maximum sensitivity substrate	Thermo Fisher Scientific	34096
Recombinant DNA		
pFA6a-mEGFP-kanMX6	Lab stock	JQW85
pFA6a-mYFP-kanMX6	Lab stock	JQW86
pFA6a-tdTomato-kanMX6	^113^	pKS392
pFA6a-tdTomato-natMX6	^113^	pKS393
pFA6a-mECitrine-kanMX6	Lab stock	JQW228
pFA6a-mNeonGreen-kanMX6	Lab stock	JQW946
pFA6a-mScarlet-I-kanMX6	Lab stock	JQW1020
Software and algorithms		
Fiji Software	^114^ PerkinElmer	RRID:SCR_002285
Volocity	Nikon	N/A
NIS Elements		N/A
