# Supplementary material for: Roles of the MO25 protein Pmo25 in contractile-ring stability and localization of the NDR kinase Sid2 during cytokinesis
Source: bioRxiv. 2025 Sep 24:2025.05.13.653815. Originally published 2025 May 14. Preprint. [Version 2] doi: 10.1101/2025.05.13.653815 (PMC12132425; doi:10.1101/2025.05.13.653815)
Supplement: 1 [file NIHPP2025.05.13.653815v2-supplement-1.pdf]

bioRxiv preprint doi: https://doi.org/10.1101/2025.05.13.653815; this version posted September 24, 2025. The copyright holder for this preprint (which was not certified by peer review) is the author/funder, who has granted bioRxiv a license to display the preprint in perpetuity. It is made available under aCC-BY 4.0 International license.

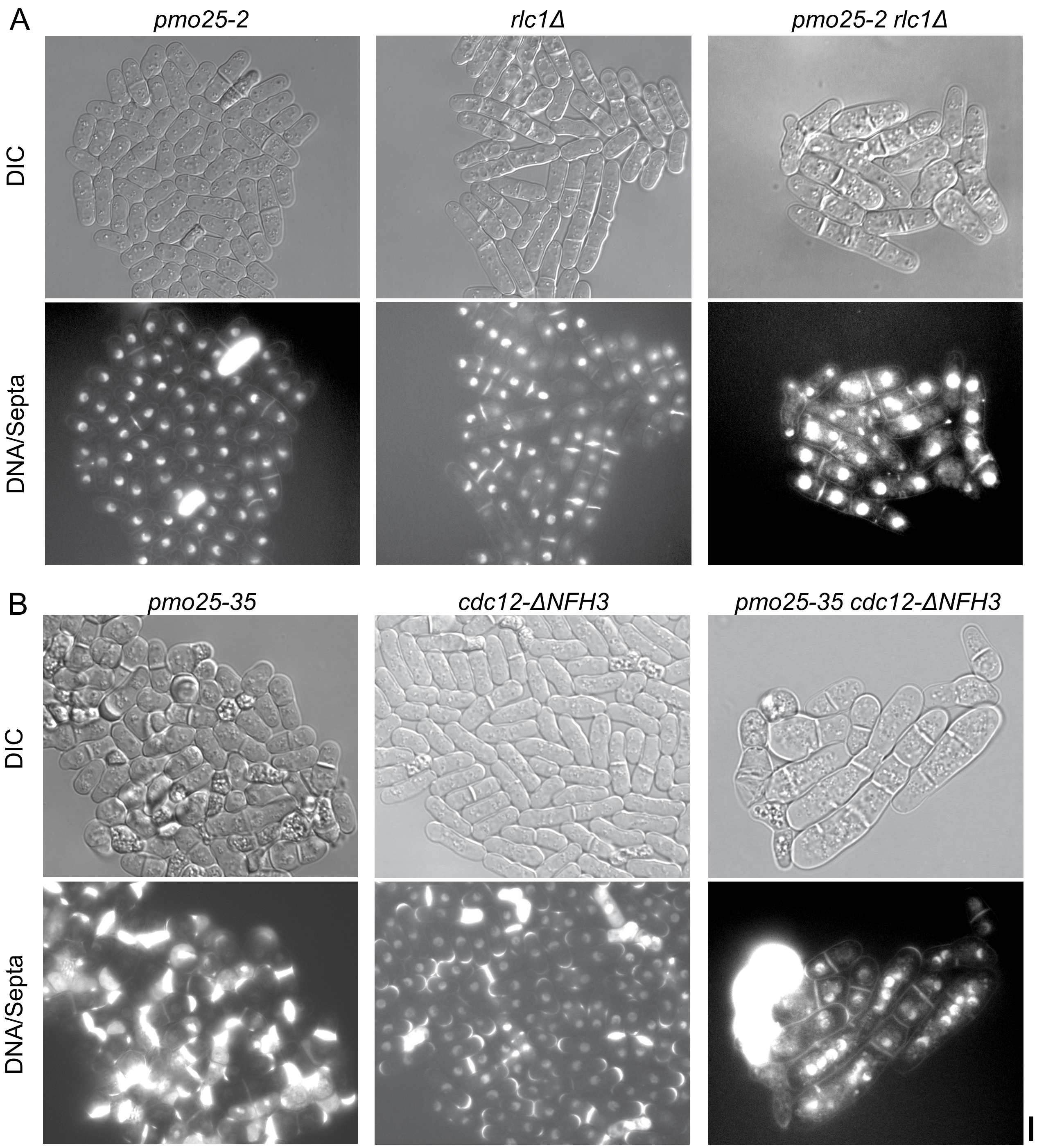

Figure S1. Synthetic genetic interactions between mutations in *pmo25* and the contractile-ring proteins myosin light chain Rlc1 (A) and formin Cdc12 (B). Related to Table 1. Images of DIC and DNA/Septa stained by the Hoechst 33258 (bisbenzimide) dye are shown. Lysed or died cells have the brightest glowing staining that obscures the neighboring cells.

(A) Cells were grown at 32°C then shifted to 25°C for 7 h before staining and imaging.

(B) Cells were grown at 25°C then shifted to 36°C for 6 h before staining and imaging. Bar, 5 µm.

bioRxiv preprint doi: https://doi.org/10.1101/2025.05.13.653815; this version posted September 24, 2025. The copyright holder for this preprint (which was not certified by peer review) is the author/funder, who has granted bioRxiv a license to display the preprint in perpetuity. It is made available under aCC-BY 4.0 International license.

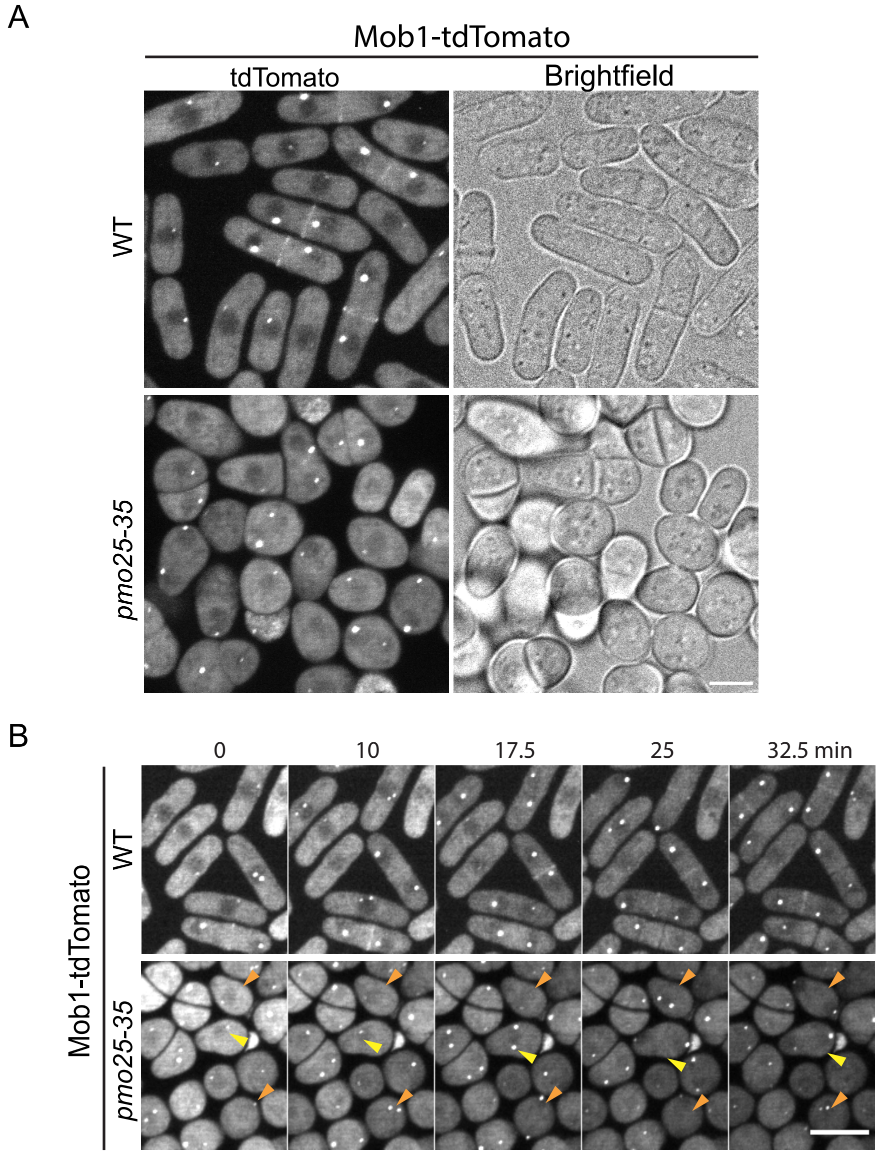

Figure S2. Pmo25 is involved in the recruitment of Mob1 to the division site. Related to Figure 4.
(A and B) Single time point images (A) and time course (in min) (B) of Mob1-tdTomato in WT (JW10137) and *pmo25-35* mutant (JW10136) grown at 36°C for 5 h before imaging at 36°C. Arrowheads mark the representative cells with weak or no Mob1 signal at the division site during cytokinesis. Bars, 5 μm.

bioRxiv preprint doi: https://doi.org/10.1101/2025.05.13.653815; this version posted September 24, 2025. The copyright holder for this preprint (which was not certified by peer review) is the author/funder, who has granted bioRxiv a license to display the preprint in perpetuity. It is made available under aCC-BY 4.0 International license.

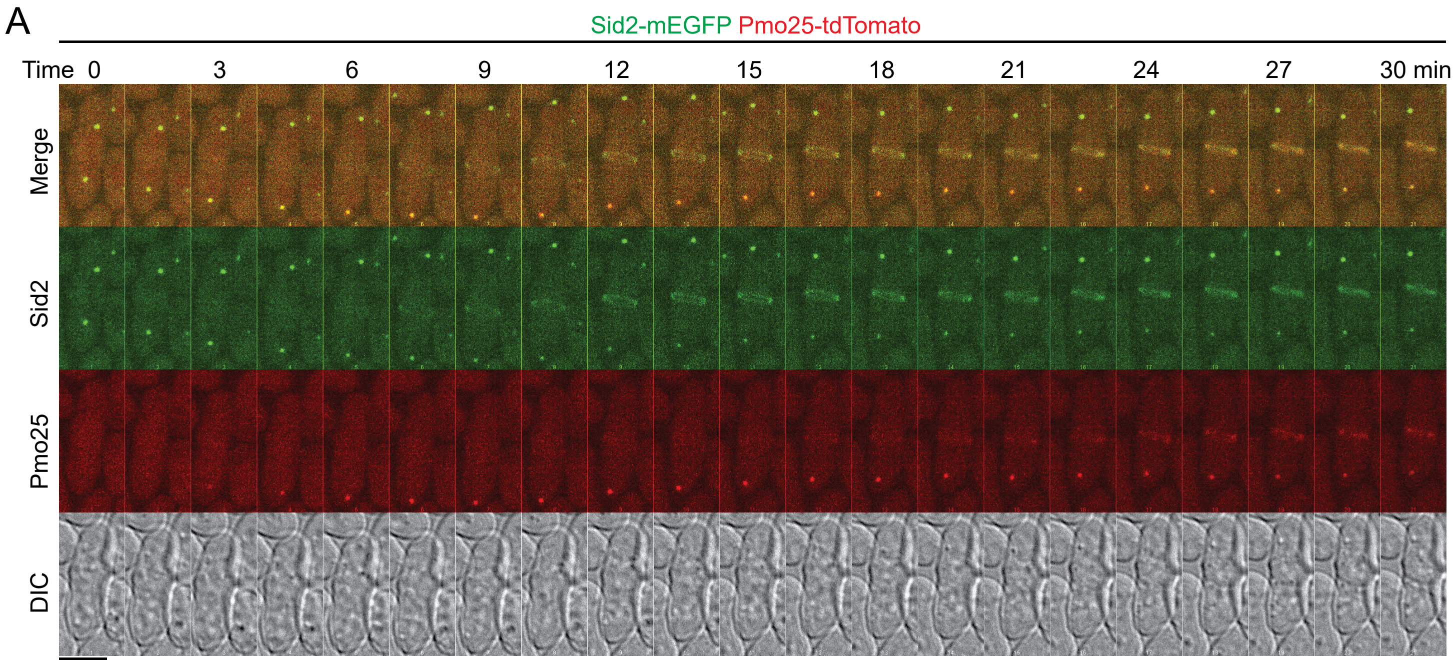

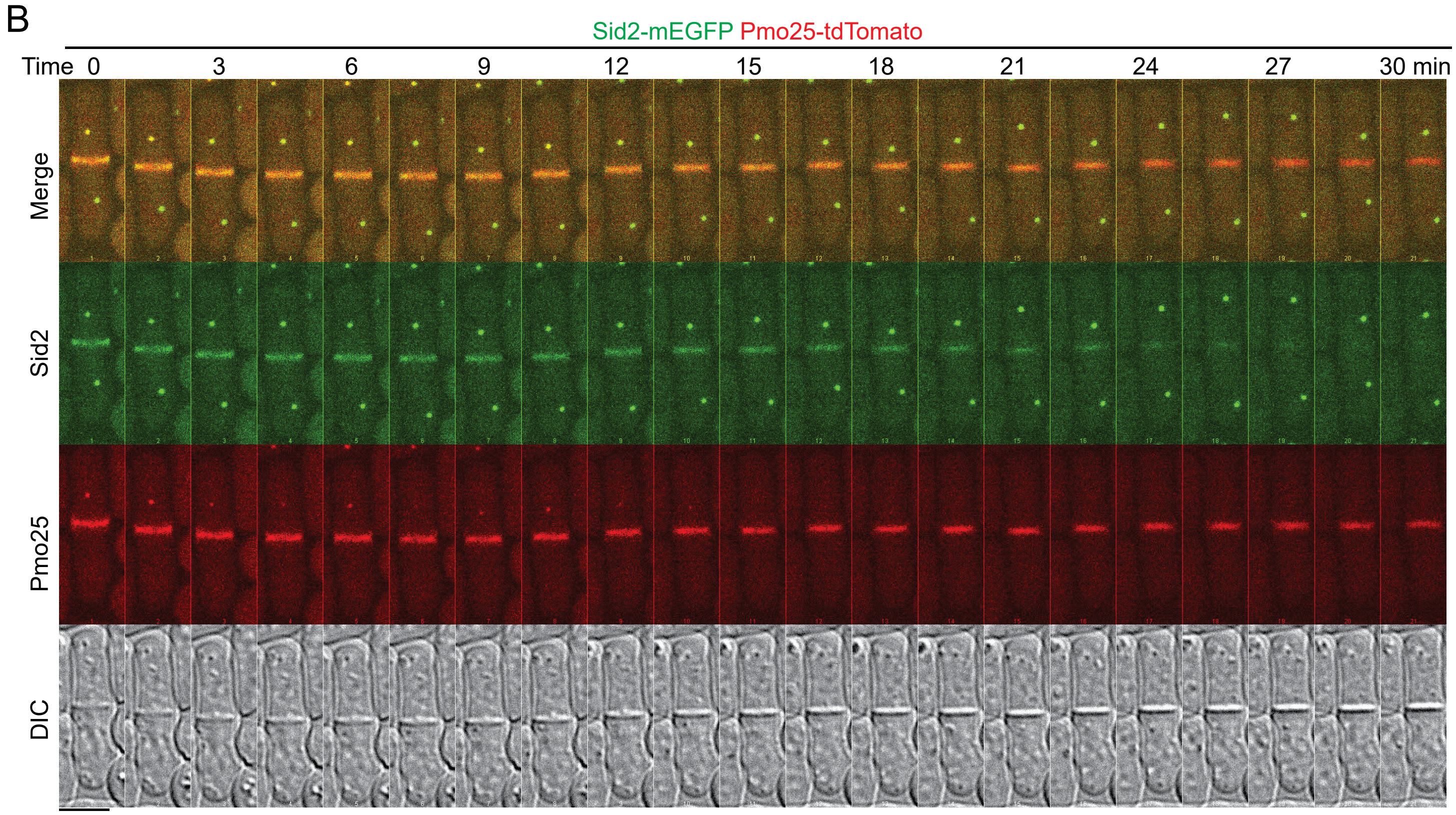

Figure S3. Time courses showing localizations of Sid2 and Pmo25 at the SPBs and the division site during cytokinesis. Related to Figure 5.
(A and B) Max intensity projections (9 slices with 0.8 μm spacing) and DIC images showing localizations of Sid2 and Pmo25 in two representative cells. Cells expressing both Sid2-mEGFP and Pmo25-tdTomato (JW10302) were imaged in time-lapse movies over 30 min. Cells were grown at 25°C for ~48 h before imaging. Bars, 5 μm.

bioRxiv preprint doi: https://doi.org/10.1101/2025.05.13.653815; this version posted September 24, 2025. The copyright holder for this preprint (which was not certified by peer review) is the author/funder, who has granted bioRxiv a license to display the preprint in perpetuity. It is made available under aCC-BY 4.0 International license.

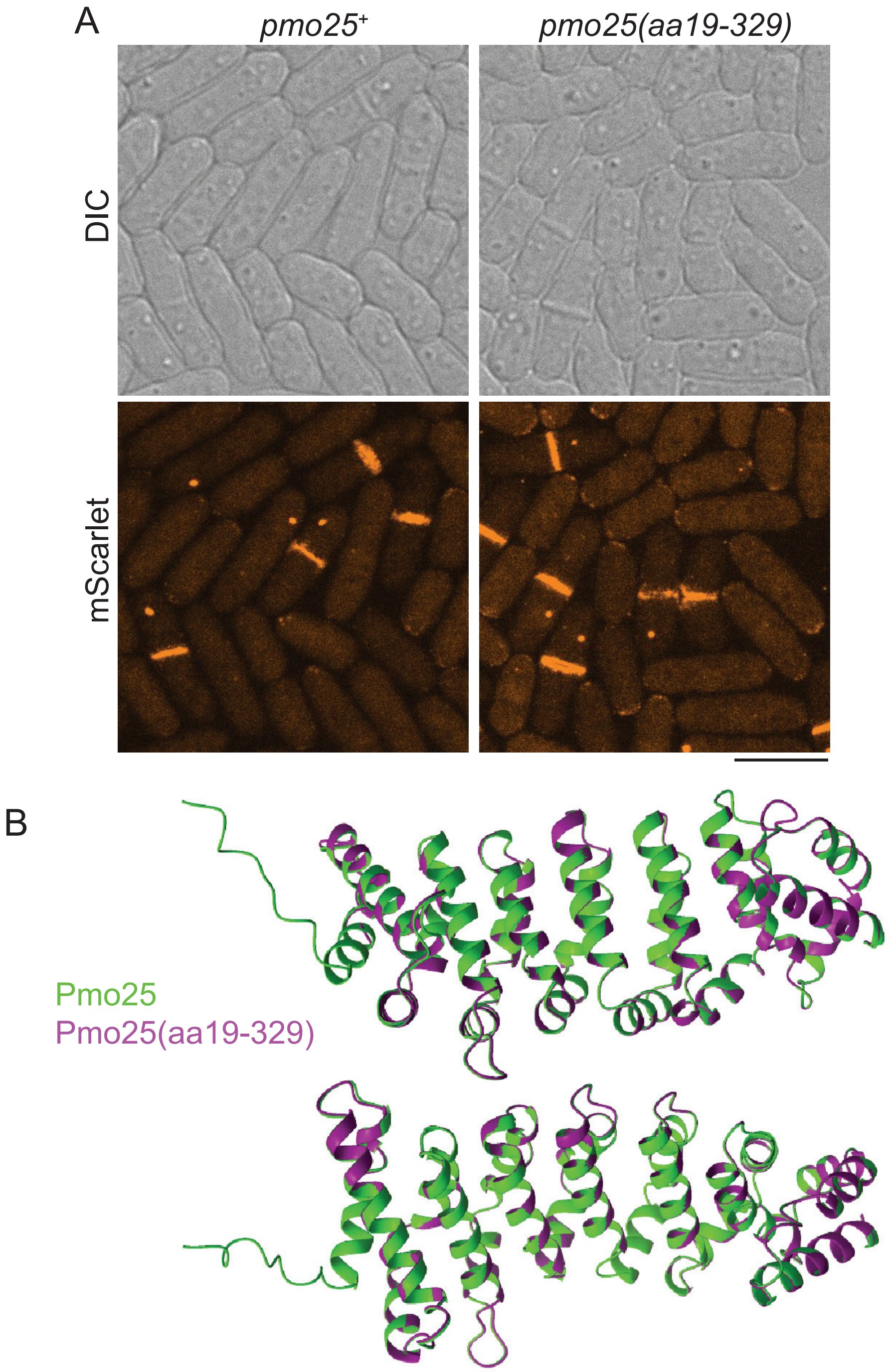

Figure S4. The morphology and localization of Pmo25-mScarlet and Pmo25(aa19-329)-mScarlet NH_2 terminal truncation mutant. Related to Figure 8.
(A) Images of cells expressing Pmo25-mScarlet (JW8034) or Pmo25(aa19-329)-mScarlet mutant (JW8894). mScarlet images are maximal intensity projection of 11 Z slices with 0.5 µm spacing. Bar, 5 µm.
(B) Two views of the overlay of the predicted structures of full length Pmo25 (green) and Pmo25(aa19-329) (purple) by AlphaFold3. The models were aligned using the Matchmaker structure analysis tool on ChimeraX.

bioRxiv preprint doi: https://doi.org/10.1101/2025.05.13.653815; this version posted September 24, 2025. The copyright holder for this preprint (which was not certified by peer review) is the author/funder, who has granted bioRxiv a license to display the preprint in perpetuity. It is made available under aCC-BY 4.0 International license.

Table S1. *S. pombe* strains used in this study. Related to the STAR methods.

Strain name	Genotype	Figure/Table/Movie/ Reference
JW8614	*wsc1-mEGFP-kanMX6 rlc1-mCherry-natMX6 sad1-mCherry-natMX6 ade6 ura4 leu1-32 his5Δ?*	Figure 1
JW8615	*pmo25-21-2-his5*$^{+}$*-kanMX6 wsc1-mEGFP-kanMX6 rlc1-mCherry-natMX6 sad1-mCherry-natMX6 ade6 ura4 leu1-32 his5Δ?*	Figure 1
JW3186	*h*$^{-}$ *kan*^{s}*-mYFP-4Gly-cdc15 Patb2-CFP-atb2 ade6 leu1-32*	Figure 1
JW8148	*pmo25-2 kan*^{s}*-mYFP-4Gly-cdc15 Patb2-CFP-atb2 ade6 leu1-32*	Figure 1, Movie 1
JW8333	*orb6-25 kan*^{s}*-mYFP-4Gly-cdc15 kan*^{s}*-Pmyo2-mCFP-myo2 Patb2-CFP-atb2 ade6 leu1-32*	Figure 1, Movie 1
JW2766	*bgs1-191 wsc1-mEGFP-kanMX6 rlc1-mCherry-natMX6 ade6-M210 leu1-32 ura4-D18 lys1*$^{+}$	Figure 1, Movie 2
JW8575	*pmo25-2 bgs1-191 wsc1-mEGFP-kanMX6 rlc1-mCherry-natMX6 ade6 leu1-32 ura4-D18 lys1*$^{+}$	Figure 1, Movie 3
	h^{+}*/h*$^{-}$ *pmo25*$^{+}$*/pmo25Δ::hphMX6 rlc1-tdTomato-natMX6/rlc1-tdTomato-natMX6 leu1*$^{+}$*::GFP-psy1/ leu1*$^{+}$*::GFP-psy1 Patb2-mRFP-atb2/Patb2-mRFP-atb2 ade6-M210/ade6-M216 ura4/ura4*	Figure 2; Movies 4 and 5
JW910	*h*$^{+}$ *kanMX6-Pcdc4-mYFP-4gly-cdc4 ade6-M210 leu1-32 ura4-D18*	Figure 3
JW8662	*pmo25-13Myc-hphMX6 ade6-M210 leu1-32 ura4-D18*	Figure 3
JW9877	*pmo25-13Myc-hphMX6 kanMX6-Pcdc4-mYFP-4Gly-cdc4 ade6-M210 leu1-32 ura4-D18*	Figure 3
JW10097	*kanMX6-Pcdc4-mYFP-4gly-cdc4 ade6-M210 ura4-D18*	Figure 3
JW10098 -2	*pmo25-35::ura4*$^{+}$ *kanMX6-Pcdc4-mYFP-4gly-cdc4 ade6-M210 leu1-32 ura4-D18*	Figure 3
JW10070	*Patb2-CFP-atb2 cdc7-YFP-kanMX6 ade6 leu1-32*	Figure 4
JW10139	*sid2-mECitrine-kanMX6 ade6 ura4-D18 leu1-32*	Figure 4; Movie 6
JW10063	*pmo25-35::ura4*$^{+}$ *Patb2-CFP-atb2 cdc7-YFP-kanMX6 ade6 leu1-32 ura4-D18*	Figure 4
JW10138	*pmo25-35::ura4*$^{+}$ *sid2-mECitrine-kanMX6 ade6 ura4-D18 leu1-32*	Figure 4; Movie 7
YDM514	*h*$^{+}$ *sid2-13Myc-kanMX6 ade6-M21X leu1-32 ura4-D18*	Figure 4 [26]
JW7943	*h*$^{-}$ *pmo25-mECitrine-kanMX6 ade6-M210 leu1-32 ura4-D18*	Figure 4
JW10082	*sid2-13Myc-kan pmo25-mECitrine-kanMX6 ade6 leu1-32 ura4-D18*	Figure 4
JW10302	*pmo25-tdTomato-natMX6 sid2-mEGFP-hphMX6 ade6 leu1-32 ura4-D18*	Figure 5; Movie 8
JW6810	*rlc1-tdTomato-natMX6 ags1Δ 3'UTR*$_{ags1}$$^{+}$*::ags1*$^{+}$*-GFP:leu1*$^{+}$*:ura4*$^{+}$*ade6 leu1-32 ura4-D18*	Figure 6
JW8677	*pmo25-21-2-his5*$^{+}$*-kanMX6 rlc1-tdTomato-natMX6 ags1Δ 3'UTR*$_{ags1}$$^{+}$*::ags1*$^{+}$*-GFP:leu1*$^{+}$*:ura4*$^{+}$ *ade6 leu1-32 ura4-D18*	Figure 6
JW5249	*GFP-bgs1-leu1*$^{+}$ *bgs1Δ::ura4*$^{+}$ *rlc1-tdTomato-natMX6 ade6-M210 leu1-32 ura4-D18*	Figure 6
JW8567	*pmo25-21-2-his5*$^{+}$*-kanMX6 GFP-bgs1-leu1*$^{+}$ *bgs1Δ::ura4*$^{+}$ *rlc1-tdTomato-natMX6 ade6 ura4 his5Δ?*	Figure 6
JW6153	*h*$^{-}$ *bgs4Δ::ura4*$^{+}$ *Pbgs4*$^{+}$*::GFP-bgs4*$^{+}$*-leu1*$^{+}$ *rlc1-tdTomato-natMX6 leu1-32 ura4-D18 his3-D1? ade6?*	Figure 6
JW8574	*pmo25-21-2-his5*$^{+}$*-kanMX6 bgs4Δ::ura4*$^{+}$ *Pbgs4*$^{+}$*::GFP-bgs4*$^{+}$*-leu1*$^{+}$ *rlc1-tdTomato-natMX6 leu1-32 ura4 his3-D1? ade6 his5Δ?*	Figure 6
JW8489	*eng1-mNeonGreen-kanMX6 ade6-210 ura4-D18 leu1-32 his5Δ?*	Figure 7

bioRxiv preprint doi: https://doi.org/10.1101/2025.05.13.653815; this version posted September 24, 2025. The copyright holder for this preprint (which was not certified by peer review) is the author/funder, who has granted bioRxiv a license to display the preprint in perpetuity. It is made available under aCC-BY 4.0 International license.

JW8488	*pmo25-21-2-his5^{+}-kanMX6 eng1-mNeonGreen-kanMX6 ade6-210 ura4 leu1-32 his5Δ?*	Figure 7
JW6655	*h^{+} ync13-13Myc-natMX6 ade6-210 leu1-32 ura4-D18*	Figure 8
JW7943	*h^{-} pmo25-mECitrine-kanMX6 ade6-M210 leu1-32 ura4-D18*	Figure 8
JW7973	*pmo25-mECitrine-kanMX6 ync13-13Myc-natMX6 ade6-M210 leu1-32 ura4-D18*	Figure 8
JW7970	*pmo25-tdTomato-natMX6 ync13-mEGFP-kanMX6 ade6-M210 leu1-32 ura4-D18*	Figure 8
JW7968	*h^{+} sad1-mCherry-natMX6 pmo25-mECitrine-kanMX6 ade6-M210 leu1-32 ura4-D18*	Figure 9
JW5814	*sad1-mCherry-natMX6 ync13-mECitrine-kanMX6 ade6-M210 leu1-32 ura4-D18*	Figure 9
JW8174	*pmo25-mECitrine-kanMX6 kan^{s}-Pmyo2-mCFP-myo2 Patb2-CFP-atb2 ade6 leu1-32 ura4-D18*	Figure 9
JW8135	*ync13-mECitrine-kanMX6 kan^{s}-Pmyo2-mCFP-myo2 ade6 leu1-32 ura4-D18?*	Figure 9
JW8396	*ync13Δ::kanMX6 pmo25-mECitrine-kanMX6 kan^{s}-Pmyo2-mCFP-myo2 Patb2-CFP-atb2 ade6 leu1-32 ura4-D18*	Figure 9
JW8136	*pmo25-2 ync13-mECitrine-kanMX6 kan^{s}-Pmyo2-mCFP-myo2 ade6 leu1-32 ura4-D18?*	Figure 9
JW864	*h^{-} rlc1Δ::kanMX6 his7-366 leu1-32 ura4-D18 ade6-M216*	Figure S1
JW8330	*pmo25-2 ade6 leu1-32 ura4-D18*	Figure S1
JW10001	*rlc1Δ::kanMX6 pmo25-2 ade6 leu1-32 ura4-D18 his7-366?*	Figure S1
JW2104	*h^{+} ura4^{+}-Pcdc12-ΔNFH3(Δ1-503)-cdc12-3YFP-kanMX6 ade6-M210 leu1-32 ura4-D18*	Figure S1
JW10028	*pmo25-35::ura4^{+} leu1-32 ura4-D18*	Figure S1
JW9994	*ura4^{+}-Pcdc12-ΔNFH3(Δ1-503)-cdc12-3YFP-kanMX6 pmo25-35::ura4^{+}ade6? leu1-32? ura4-D18*	Figure S1
JW10136	*pmo25-35::ura4^{+} mob1-tdTomato-kanMX6 ade6 ura4-D18 leu1-32*	Figure S2
JW10137	*mob1-tdTomato-kanMX6 ade6 ura4-D18 leu1-32*	Figure S2
JW10302	*pmo25-tdTomato-natMX6 sid2-mEGFP-hphMX6 ade6 leu1-32 ura4-D18*	Figure S3
JW8034	*h^{-} pmo25-mScarlet-I-kanMX6 ade6-M210 leu1-32 ura4-D18*	Figure S4
JW8894	*natMX6-Ppmo25-pmo25(aa19-329)-mScarlet-I-kanMX6 ade6-M210 leu1-32 ura4-D18*	Figure S4
JW81	*h^{-} ade6-210 leu1-32 ura4-D18*	Table 1
JW8330	*pmo25-2 ade6 leu1-32 ura4-D18*	Table 1
JW8307	*pmo25-20-his5^{+}-kanMX6 ade6-M210 leu1-32 ura4*	Table 1
JW8435	*pmo25-21-2-his5^{+}-kanMX6 ade6-210 ura4 leu1-32 his5Δ?*	Table 1
JW10028	*pmo25-35::ura4^{+} leu1-32 ura4-D18*	Table 1
JW864	*h^{-} rlc1Δ::kanMX6 his7-366 leu1-32 ura4-D18 ade6-M216*	Table 1
JW10001	*rlc1Δ::kanMX6 pmo25-2 ade6 leu1-32 ura4-D18 his7-366?*	Table 1
JW10002	*rlc1Δ::kanMX6 pmo25-20-his5^{+}-kanMX6 ade6 leu1-32 ura4-D18 his7-366?*	Table 1
JW10000	*rlc1Δ::kanMX6 pmo25-21-2-his5^{+}-kanMX6 ade6 leu1-32 ura4-D18 his5Δ? his7-366?*	Table 1
JW10031	*pmo25-35::ura4^{+} rlc1Δ::kanMX6 his7-366? leu1-32 ura4-D18 ade6-M216?*	Table 1

bioRxiv preprint doi: https://doi.org/10.1101/2025.05.13.653815; this version posted September 24, 2025. The copyright holder for this preprint (which was not certified by peer review) is the author/funder, who has granted bioRxiv a license to display the preprint in perpetuity. It is made available under aCC-BY 4.0 International license.

MBY925	*h⁻ myo2-ΔIQ1ΔIQ2::ura4⁺ leu1-32 ura4-D18*	Table 1[129]
JW9989	*myo2-ΔIQ1ΔIQ2::ura4⁺ pmo25-2 leu1-32 ura4-D18 ade6?*	Table 1
MBY53	*h⁻ myo2-S1 ade6-M21x ura4-D18 leu1-32*	Table 1[130]
JW10009	*pmo25-2 myo2-S1 ade6 leu1-32 ura4-D18*	Table 1
JW10013	*pmo25-21-2-his5⁺-kanMX6 myo2-S1 ade6 leu1-32 ura4-D18 his5Δ?*	Table 1
MBY54	*h⁻ myo2-S2 ade6-M21x ura4-D18*	Table 1[130]
JW10011	*pmo25-2 myo2-S2 ade6 leu1-32 ura4-D18*	Table 1
JW10012	*pmo25-21-2-his5⁺-kanMX6 myo2-S2 ade6 leu1-32 ura4-D18 his5Δ?*	Table 1
JW2252	*myo2-E1 ade6 leu1-32 ura4-D18*	Table 1
JW10010	*pmo25-2 myo2-E1 ade6 leu1-32 ura4-D18*	Table 1
JW2104	*h⁺ ura4⁺-Pcdc12-ΔNFH3(Δ1-503)-cdc12-3YFP-kanMX6 ade6-M210 leu1-32 ura4-D18*	Table 1
JW9993	*ura4⁺-Pcdc12-ΔNFH3(Δ1-503)-cdc12-3YFP-kanMX6 pmo25-2 ade6 leu1-32 ura4-D18*	Table 1
JW9994	*ura4⁺-Pcdc12-ΔNFH3(Δ1-503)-cdc12-3YFP-kanMX6 pmo25-35::ura4⁺ade6? leu1-32? ura4-D18*	Table 1
JW2249	*rng2-346 ade6-M210 leu1-32 ura4-D18*	Table 1
JW10014	*pmo25-2 rng2-346 ade6 leu1-32 ura4-D18*	Table 1
JW994	*h⁻ cdc4-8 his7-366 leu1-32 ura4-D18 ade6-M216*	Table 1
JW9986	*cdc4-8 pmo25-2 leu1-32 ura4-D18 ade6 his7-366?*	Table 1
JW10003	*cdc4-8 pmo25-21-2-his5⁺-kanMX6 ade6 leu1-32 ura4-D18 his5Δ? his7-366?*	Table 1

bioRxiv preprint doi: https://doi.org/10.1101/2025.05.13.653815; this version posted September 24, 2025. The copyright holder for this preprint (which was not certified by peer review) is the author/funder, who has granted bioRxiv a license to display the preprint in perpetuity. It is made available under aCC-BY 4.0 International license.

Movie legends

Movie 1. Contractile-ring defects in *orb6-25* and *pmo25-2* cells. Related to Figure 1C. Cells of *orb6-25 mYFP-cdc15 CFP-atb2 CFP-myo2* (JW8333, left) and *pmo25-2 mYFP-cdc15 CFP-atb2* (JW8148, right) were grown in log phase at 25°C for ~36 h and then shifted to 36°C for 8 h before imaging at 36°C with 2 min interval. The movie shows the maximal intensity projection of 17 Z slices with 0.5 um spacing. Green: Cdc15; Red: Atb2 Myo2 or Atb2. 7 frames per second (FPS).

Movie 2. Contractile rings in *bgs1-191* cells. Related to Figures 1D and 1E. Cells expressing Wsc1-mEGFP Rlc1-mCherry in *bgs1/cps1-191* were grown in log phase at 25°C for ~36 h and then shifted to 36°C for 2 h before imaging at ~36°C with 2 min interval. The movie only showed the Rlc1-mCherry channel at maximal intensity projection of 9 Z slices with 0.75 μm spacing. 4 FPS. Bar, 5 μm.

Movie 3. Contractile rings in *pmo25-2 bgs1-191* cells. Related to Figures 1D and 1E. Cells expressing Wsc1-mEGFP Rlc1-mCherry in *pmo25-2 bgs1-191* mutant were grown in log phase at 25°C for ~36 h and then shifted to 36°C for 2 h before imaging at ~36°C with 2 min interval. The movie only showed the Rlc1-mCherry channel at maximal intensity projection of 9 Z slices with 0.75 μm spacing. 4 FPS. Bar, 5 μm.

Movie 4. Cytokinesis in *pmo25*$^+$ cells. Related to Figure 2. Cells expressing GFP-Psy1 (green) and Rlc1-tdTomato mRFP-Atb2 (red) in *pmo25*$^+$ WT were grown at 25°C after tetrad dissection as in Figure 2B. Time lapse movie was performed with 8 min interval. Maximal intensity projection of 7 Z slices with 1.0 μm spacing was shown. 2 FPS. Bar, 5 μm.

Movie 5. Cytokinesis in *pmo25Δ* cells. Related to Figure 2. Cells expressing GFP-Psy1 (green) and Rlc1-tdTomato mRFP-Atb2 (red) in *pmo25Δ* cells were grown at 25°C after tetrad dissection as in Figure 2B. Time lapse movie was performed with 8 min interval. Maximal intensity projection of 7 Z slices with 1.0 μm spacing was shown. 2 FPS. Bar, 5 μm.

Movie 6. Sid2-mECitrine localization in WT (*pmo25*$^+$) cells. Related to Figures 4B-4E. Cells expressing Sid2-mECitrine in *pmo25*$^+$ WT were grown in log phase at 25C°C for ~36 h, then shifted to 36°C for 4 h before imaging at 36°C. Time lapse movie was performed with 2.5 min interval. Maximal intensity projection of 9 Z slices with 0.75 μm spacing was shown. 1 FPS. Bar, 5 μm.

Movie 7. Sid2-mECitrine localization in *pmo25-35* cells. Related to Figures 4B-4E. Cells expressing Sid2-mECitrine in *pmo25-35* were grown in log phase at 25C°C for ~36 h, then shifted to 36°C for 4 h before imaging at 36°C. Time lapse movie was performed with 2.5 min interval. Maximal intensity projection of 9 Z slices with 0.75 μm spacing was shown. 1 FPS. Bar, 5 μm.

Movie 8. Sid2-mEGFP and Pmo25-tdTomato colocalization at the SPBs and the division site. Related to Figures 5 and S3. Cells expressing both Sid2-mEGFP and Pmo25-tdTomato (JW10302) were grown at 25°C for ~48 h before imaging. Time lapse movies were performed with 1.5 min intervals. Maximal intensity projection of 9 Z slices with 0.8 μm spacing was shown. DIC (top left), GFP (top right), tdTomato (bottom left), merge (bottom right). 2 FPS.
