# Supplementary material for: Roles of the MO25 protein Pmo25 in contractile-ring stability and localization of the NDR kinase Sid2 during cytokinesis
Source: bioRxiv. 2025 May 14:2025.05.13.653815. Preprint. [Version 1] doi: 10.1101/2025.05.13.653815 (PMC12132425; doi:10.1101/2025.05.13.653815)

bioRxiv preprint doi: https://doi.org/10.1101/2025.05.13.653815; this version posted May 14, 2025. The copyright holder for this preprint (which was not certified by peer review) is the author/funder, who has granted bioRxiv a license to display the preprint in perpetuity. It is made available under aCC-BY 4.0 International license.

Table S1. *S. pombe* strains used in this study. Related to the STAR methods.

Strain name	Genotype	Figure/reference
JW6655	*h^+ ync13-13Myc-natMX6 ade6-210 leu1-32 ura4-D18*	Figure 1
JW7943	*h^- pmo25-mECitrine-kanMX6 ade6-M210 leu1-32 ura4-D18*	Figure 1
JW7973	*pmo25-mECitrine-kanMX6 ync13-13Myc-natMX6 ade6-M210 leu1-32 ura4-D18*	Figure 1
JW7970	*pmo25-tdTomato-natMX6 ync13-mEGFP-kanMX6 ade6-M210 leu1-32 ura4-D18*	Figure 1
JW8089	*pmo25-mScarlet-I-kanMX6 ync13-mECitrine-kanMX6 ade6-M210 leu1-32 ura4-D18*	Figure 1
JW7968	*h^+ sad1-mCherry-natMX6 pmo25-mECitrine-kanMX6 ade6-M210 leu1-32 ura4-D18*	Figure 2
JW5814	*sad1-mCherry-natMX6 ync13-mECitrine-kanMX6 ade6-M210 leu1-32 ura4-D18*	Figure 2
JW8174	*pmo25-mECitrine-kanMX6 kan^s-Pmyo2-mCFP-myo2 Patb2-CFP-atb2 ade6 leu1-32 ura4-D18*	Figure 2
JW8135	*ync13-mECitrine-kanMX6 kan^s-Pmyo2-mCFP-myo2 ade6 leu1-32 ura4-D18?*	Figure 2
JW8396	*ync13Δ::kanMX6 pmo25-mECitrine-kanMX6 kan^s-Pmyo2-mCFP-myo2 Patb2-CFP-atb2 ade6 leu1-32 ura4-D18*	Figure 2
JW8136	*pmo25-2 ync13-mECitrine-kanMX6 kan^s-Pmyo2-mCFP-myo2 ade6 leu1-32 ura4-D18?*	Figure 2
JW3186	*h^- kan^s mYFP-4Gly-cdc15 Patb2-CFP-atb2 ade6 leu1-32*	Figure 3
JW8148	*pmo25-2 kans mYFP-4Gly-cdc15 Patb2-CFP-atb2 ade6 leu1-32*	Figure 3
JW2766	*bgs1-191 wsc1-mEGFP-kanMX6 rlc1-mCherry-natMX6 ade6-M210 leu1-32 ura4-D18 lys1-131?*	Figure 3
JW8575	*pmo25-2 bgs1-191 wsc1-mEGFP-kanMX6 rlc1-mCherry-natMX6 ade6 leu1-32 ura4-D18 lys1-131?*	Figure 3
JW8615	*pmo25-21-2-$his5^+$-kanMX6 wsc1-mEGFP-kanMX6 rlc1-mCherry-natMX6 sad1-mCherry-natMX6 ade6 ura4 leu1-32 his5Δ?*	Figure 3
JW8614	*wsc1-mEGFP-kanMX6 rlc1-mCherry-natMX6 sad1-mCherry-natMX6 ade6 ura4 leu1-32 his5Δ?*	Figure 3
	h^+/h^- $pmo25^+$/pmo25Δ::hphMX6 rlc1-tdTomato-natMX6/rlc1-tdTomato-natMX6 $leu1^+$::GFP-psy1/ $leu1^+$::GFP-psy1 Patb2-mRFP-atb2/Patb2-mRFP-atb2 ade6-M210/ade6-M216 ura4/ura4	Figure 4
JW910	*h^+ kanMX6-Pcdc4-mYFP-4gly-cdc4 ade6-M210 leu1-32 ura4-D18*	Figure 5
JW8662	*pmo25-13Myc-hphMX6 ade6-M210 leu1-32 ura4-D18*	Figure 5
JW9877	*pmo25-13Myc-hphMX6 kanMX6-Pcdc4-mYFP-4Gly-cdc4 ade6-M210 leu1-32 ura4-D18*	Figure 5
JW10097	*kanMX6-Pcdc4-mYFP-4gly-cdc4 ade6-M210 ura4-D18*	Figure 5
JW10098-2	*pmo25-35::$ura4^+$ kanMX6-Pcdc4-mYFP-4gly-cdc4 ade6-M210 leu1-32 ura4-D18*	Figure 5
JW10070	*Patb2-CFP-atb2 cdc7-YFP-kanMX6 ade6 leu1-32*	Figure 6
JW10139	*sid2-mECitrine-kanMX6 ade6 ura4-D18 leu1-32*	Figure 6
JW10063	*pmo25-35::$ura4^+$ Patb2-CFP-atb2 cdc7-YFP-kanMX6 ade6 leu1-32 ura4-D18*	Figure 6
JW10138	*pmo25-35::$ura4^+$ sid2-mECitrine-kanMX6 ade6 ura4-D18 leu1-32*	Figure 6

bioRxiv preprint doi: https://doi.org/10.1101/2025.05.13.653815; this version posted May 14, 2025. The copyright holder for this preprint (which was not certified by peer review) is the author/funder, who has granted bioRxiv a license to display the preprint in perpetuity. It is made available under aCC-BY 4.0 International license.

YDM514	h^{+} sid2-13Myc::kanMX6 ade6-M21X leu1-32 ura4-D18	Figure 6 [26]
JW7943	h^{-} pmo25-mECitrine-kanMX6 ade6-M210 leu1-32 ura4-D18	Figure 6
JW10082	sid2-13myc::kan pmo25-mECitrine-kanMX6 ade6 leu1-32 ura4-D18	Figure 6
JW10302	pmo25-tdTomato-natMX6 sid2-mEGFP-hphMX6 ade6 leu1-32 ura4-D18	Figure 7
JW5429	gef2Δ::hphMX6 imp2::$ura4^{+}$ rlc1-tdTomato-natMX6 sad1-mEGFP-kanMX6 ade6-M21X leu1-32 ura4-D18	Figure 8
JW6153	h- bgs4Δ::$ura4^{+}$ P$bgs4^{+}$::GFP-$bgs4^{+}$-$leu1^{+}$ rlc1-tdTomato-natMX6 leu1-32 ura4-D18 his3-D1? ade6?	Figure 8
JW6810	rlc1-tdTomato-natMX6 ags1Δ 3'$UTR_{ags1}{}^{+}$::$ags1^{+}$-GFP:$leu1^{+}$:$ura4^{+}$ade6 leu1-32 ura4-D18	Figure 8
JW8567	GFP-bgs1-$leu1^{+}$ bgs1Δ::$ura4^{+}$ pmo25-21-2-$his5^{+}$-kanMX6 rlc1-tdTomato-natMX6 ade6 ura4 his5Δ?	Figure 8
JW8574	pmo25-21-2-$his5^{+}$-kanMX6 bgs4Δ::$ura4^{+}$ P$bgs4^{+}$::GFP-$bgs4^{+}$-$leu1^{+}$ rlc1-tdTomato-natMX6 leu1-32 ura4 his3-D1? ade6 his5Δ?	Figure 8
JW8677	rlc1-tdTomato-natMX6 ags1Δ 3'$UTR_{ags1}{}^{+}$::$ags1^{+}$-GFP:$leu1^{+}$:$ura4^{+}$ade6 leu1-32 ura4	Figure 8
JW8489	eng1-mNeonGreen-kanMX6 ade6-210 ura4-D18 leu1-32 his5Δ?	Figure 9
JW8488	pmo25-21-2-$his5^{+}$-kanMX6 eng1-mNeonGreen-kanMX6 ade6-210 ura4 leu1-32 his5Δ?	Figure 9
JW10136	pmo25-35::$ura4^{+}$ mob1-tdTomato-kanMX6 ade6 ura4-D18 leu1-32	Figure S1
JW10137	mob1-tdTomato-kanMX6 ade6 ura4-D18 leu1-32	Figure S1
JW10302	pmo25-tdTomato-natMX6 sid2-mEGFP-hphMX6 ade6 leu1-32 ura4-D18	Figure S2

bioRxiv preprint doi: https://doi.org/10.1101/2025.05.13.653815; this version posted May 14, 2025. The copyright holder for this preprint (which was not certified by peer review) is the author/funder, who has granted bioRxiv a license to display the preprint in perpetuity. It is made available under aCC-BY 4.0 International license.

Figure S1. Pmo25 is involved in Mob1's recruitment to the division site.
(A and B) Single time point images (A) and time course (in min) (B) of Mob1-tdTomato in WT (JW10137) and *pmo25-35* mutant (JW10136) grown at 36°C for 5 h before imaging at 36°C. Bars, 5 μm.

Figure S2. Time courses showing localizations of Sid2 and Pmo25 at the SPBs and the division site during cytokinesis.
(A and B) Max projections and DIC images showing localizations of Sid2 and Pmo25 over 30 min. Cells expressing both Sid2-mEGFP and Pmo25-tdTomato (JW10302) were imaged in time-lapse movies. Maximal intensity projections of 9 slices with 0.8 µm spacing were shown. Cells were grown at 25°C for ~48 h before imaging. Two representative cells are shown. Bars, 5 µm.

Movie legends

Movie 1. Cytokinesis in *bgs1-191*. Cells expressing Wsc1-mEGFP Rlc1-mCherry in *bgs1/cps1-191* were grown in log phase at 25°C for ~36 h and then shifted to 36°C for 2 h before imaging at ~36°C with 2 min interval. The movie only showed Rlc1-mCherry channel at maximal intensity projection of 9 Z slices with 0.75 μm spacing. 4 frame per second (FPS). Bar, 5 μm.

Movie 2. Cytokinesis in *pmo25-2 bgs1-191*. Cells expressing Wsc1-mEGFP Rlc1-mCherry in *pmo25-2 bgs1-191* mutant were grown in log phase at 25°C for ~36 h and then shifted to 36°C for 2 h before imaging at ~36°C with 2 min interval. The movie only showed Rlc1-mCherry channel at maximal intensity projection of 9 Z slices with 0.75 μm spacing. 4 FPS. Bar, 5 μm.

Movie 3. Cytokinesis in *pmo25*$^{+}$. Cells expressing GFP-Psy1 Rlc1-tdTomato RFP-Atb2 in *pmo25*$^{+}$ WT were grown at 25°C after tetrad dissection as in Figure 4B. Time lapse movie was

bioRxiv preprint doi: https://doi.org/10.1101/2025.05.13.653815; this version posted May 14, 2025. The copyright holder for this preprint (which was not certified by peer review) is the author/funder, who has granted bioRxiv a license to display the preprint in perpetuity. It is made available under aCC-BY 4.0 International license.

performed with 8 min interval. Maximal intensity projection of 7 Z slices with 1.0 μm spacing was shown. 2 FPS. Bar, 5 μm.

Movie 4. Cytokinesis in *pmo25Δ*. Cells expressing GFP-Psy1 Rlc1-tdTomato RFP-Atb2 in *pmo25Δ* were grown at 25°C after tetrad dissection as in Figure 4B. Time lapse movie was performed with 8 min interval. Maximal intensity projection of 7 Z slices with 1.0 μm spacing was shown. 2 FPS. Bar, 5 μm.

Movie 5. Sid2-mECitrine localization in WT (*pmo25⁺*). Cells expressing Sid2-mECitrine in *pmo25⁺* WT were grown in log phase at 25C°C for ~36 h, then shifted to 36°C for 4 h before imaging at 36°C. Time lapse movie was performed with 2.5 min interval. Maximal intensity projection of 9 Z slices with 0.75 μm spacing was shown. 1 FPS. Bar, 5 μm.

Movie 6. Sid2-mECitrine localization in *pmo25-35* cells. Cells expressing Sid2-mECitrine in *pmo25-35* were grown in log phase at 25C°C for ~36 h, then shifted to 36°C for 4 h before imaging at 36°C. Time lapse movie was performed with 2.5 min interval. Maximal intensity projection of 9 Z slices with 0.75 μm spacing was shown. 1 FPS. Bar, 5 μm.

Movie 7. Sid2-mEGFP and Pmo25-tdTomato colocalization at the SPBs and the division site. Cells expressing both Sid2-mEGFP and Pmo25-tdTomato (JW10302) were grown at 25°C for ~48 h before imaging. Time lapse movies were performed with 1.5 min intervals. Maximal intensity projection of 9 Z slices with 0.8 µm spacing was shown. 2 FPS. DIC (top left), GFP (top right), tdTomato (bottom left), merge of GFP and tdTomato (bottom right).

bioRxiv preprint doi: https://doi.org/10.1101/2025.05.13.653815; this version posted May 14, 2025. The copyright holder for this preprint (which was not certified by peer review) is the author/funder, who has granted bioRxiv a license to display the preprint in perpetuity. It is made available under aCC-BY 4.0 International license.

Supple Figure 1

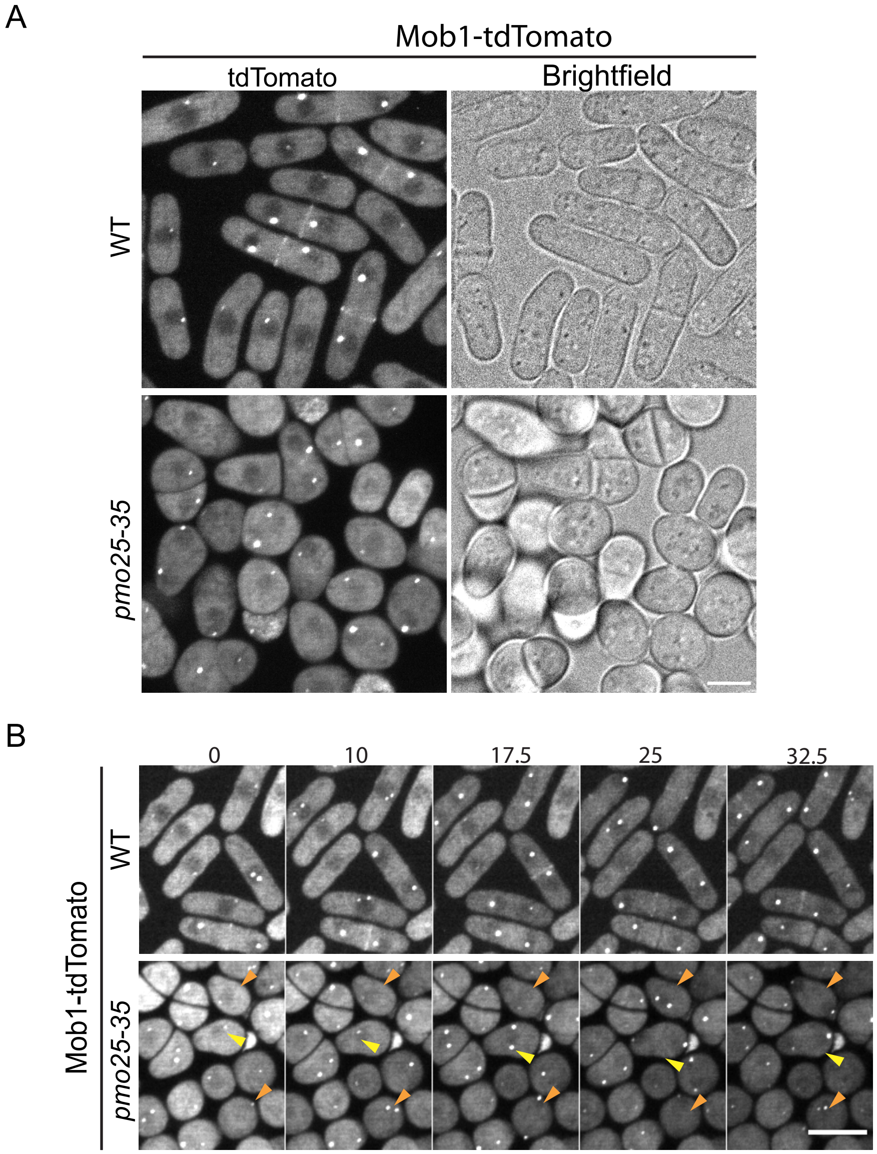

bioRxiv preprint doi: https://doi.org/10.1101/2025.05.13.653815; this version posted May 14, 2025. The copyright holder for this preprint (which was not certified by peer review) is the author/funder, who has granted bioRxiv a license to display the preprint in perpetuity. It is made available under aCC-BY 4.0 International license.

Ye et al., Figure S2

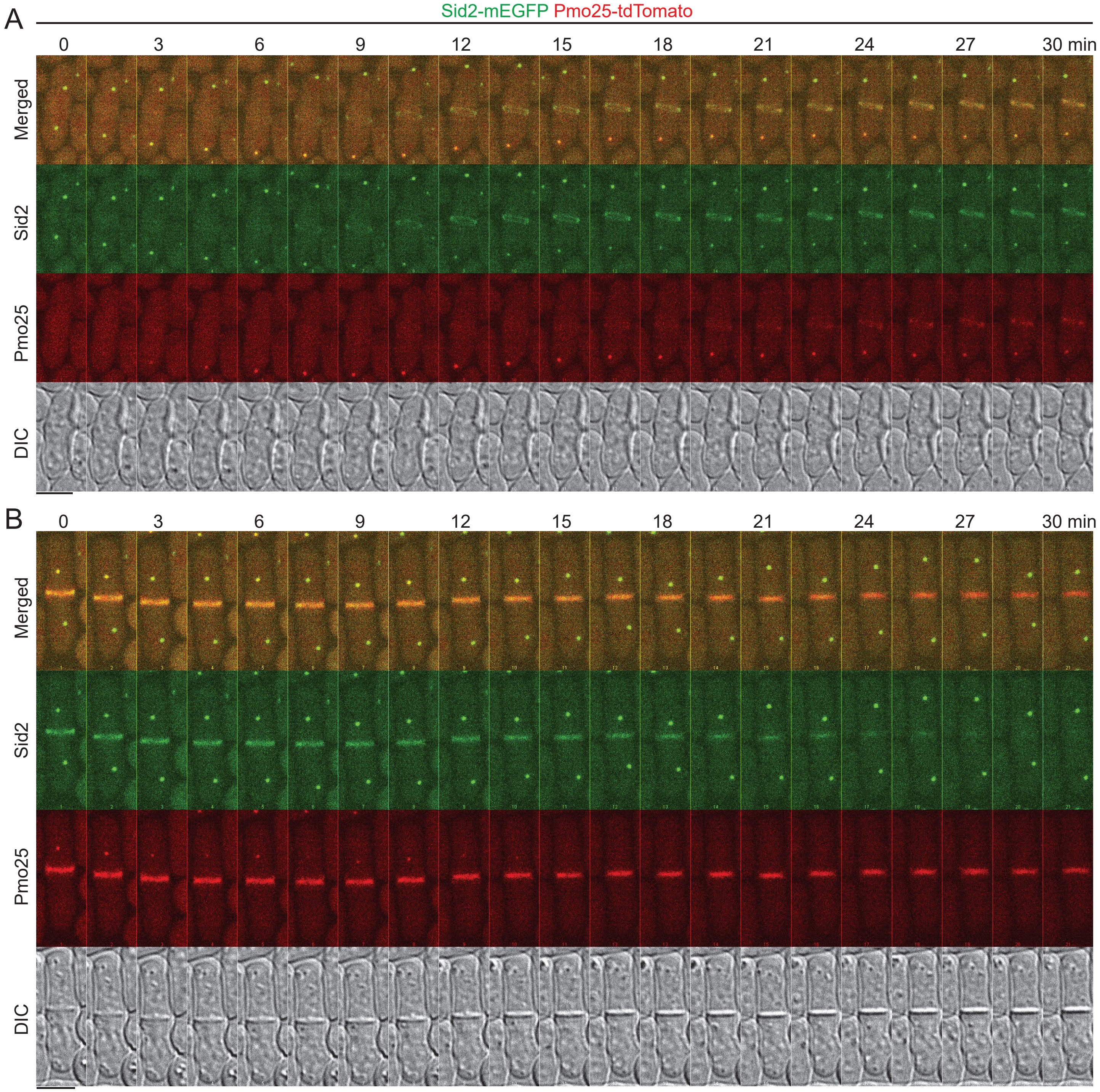

Supplement: Supplementary file 1 [file NIHPP2025.05.13.653815v1-supplement-1.pdf]
